# The effect of surface nucleation modulation on the mechanical and biocompatibility of metal-polymer biomaterials

**DOI:** 10.3389/fbioe.2023.1160351

**Published:** 2023-04-06

**Authors:** Zhenhong Ye, Le Zhang, Taiwei Liu, Weicheng Xuan, Xiaodong He, Changhao Hou, Donglin Han, Binbin Yu, Junye Shi, Jie Kang, Jiangping Chen

**Affiliations:** ^1^ Institute of Refrigeration and Cryogenics, School of Mechanical Engineering, Shanghai Jiao Tong University, Shanghai, China; ^2^ Shanghai High Efficiency Cooling System Research Center, Shanghai, China; ^3^ State Key Laboratory for Oncogenes and Bio-ID Center, School of Biomedical Engineering, Shanghai Jiao Tong University, Shanghai, China; ^4^ Department of Engineering Mechanics, School of Naval Architecture, Ocean and Civil Engineering, Shanghai Jiao Tong University, Shanghai, China; ^5^ Department of Engineering Mechanics and Innovation Center for Advanced Ship and Deep-Sea Exploration, School of Naval Architecture, Ocean and Civil Engineering Shanghai Jiao Tong University, Shanghai, China; ^6^ Department of Urology, Shanghai Sixth People’s Hospital Affiliated to Shanghai Jiao Tong University School of Medicine, Shanghai, China; ^7^ Department of General Surgery, Sixth People’s Hospital, Shanghai Jiao Tong University, Shanghai, China

**Keywords:** nucleation analysis, biomechanics, hernia, hyperviscoelasticity, valence bond analysis

## Abstract

The deployment of hernia repair patches in laparoscopic procedures is gradually increasing. In this technology, however, understanding the new phases of titanium from the parent phase on polymer substrates is essential to control the microstructural transition and material properties. It remains a challenging area of condensed matter physics to predict the kinetic and thermodynamic properties of metals on polymer substrates from the molecular scale due to the lack of understanding of the properties of the metal-polymer interface. However, this paper revealed the mechanism of nucleation on polymer substrates and proposed for the first record a time-dependent regulatory mechanism for the polymer-titanium interface. The interconnection between polymer surface chain entanglement, nucleation and growth patterns, crystal structure and surface roughness were effectively unified. The secondary regulation of mechanical properties was accomplished simultaneously to satisfy the requirement of biocompatibility. Titaniumized polypropylene patches prepared by time-dependent magnetron sputtering technology demonstrated excellent interfacial mechanical properties and biocompatibility. In addition, modulation by low-temperature plasma metal deposition opened a new pathway for biomaterials. This paper provides a solid theoretical basis for the research of titanium nanofilms on medical polypropylene substrates and the medical industry of implantable biomaterials, which will be of great value in the future.

## Introduction

The treatment of inguinal and incisional hernias is growing exponentially with an ageing population (Currie et al., 2021). Although commonly used polypropylene polymers have elastic and viscous mechanical properties close to those of living organisms, they lack surface properties. This often results in poor biocompatibility and the requirement for biocompatible barrier coating treatments to address the bio-inflammatory response after their implantation. The control of phase changes involved in coating preparation is widely used in the design and processing of materials. This is essential for modulating the microstructure to obtain specific desired properties such as surface adsorption and transport, catalytic activity and optical properties (Meiners et al., 2020; Yuan et al., 2022). Classical nucleation and growth process begin with the structural transformation of a new phase in the parent phase. Two influencing factors of the nucleation process, that is, the interfacial energy and the thermodynamically based Brownian motion of the particles, are of great importance. There is one or more intermediate states before the evolution to the final equilibrium product phase. They differ distinctly from the product phase in terms of composition, crystal structure and chemical order (Ou et al., 2020). The nucleation potential of this series of sub-stable intermediates is small compared to that of the stable phase. Eventually nucleation of stable phases evolves from the sub-stable phases, or heterogeneously occurs on sub-stable nanoclusters. The study of “missing links” that span the intermediate structure between the atom and solid crystalline phases contributes to the understanding of crystal evolution (Sadeghi et al., 2015). The dominance of surface nucleation and the intrinsic diffusion coefficient lead to contradictory nucleation and growth phenomena (Thiyaneshwaran et al., 2018). This has an impact on both lattice structure and grain growth. The long-term stability, mechanical properties and creep behavior of the material interface are largely dependent on these properties. At the same time, interparticle interactions guide the approaching new particles to find energetically favorable directions for attachment to the crystal (Zhang et al., 2014). The surface morphology in the crystalline system significantly alters the crystallization pathway due to the reduced nucleation barriers resulting from the reduction in interfacial free energy (Dey et al., 2010). Non-classical nucleation studies of inorganic nanoparticles in solution, protein crystallization, diamond crystal nucleation and crystallization in the amorphous states have been reported (Myerson and Trout, 2013; Lupulescu and Rimer, 2014; Houben et al., 2020). However, the study of crystallization by particle attachment (CPA) has been mainly restricted to inorganic systems. The atomic details relating to the complex reactions involved in the particle-attached crystallization of metals on polymer surfaces remain elusive. As a result, predictive descriptions linking molecular details to overall behavior are still lacking (De Yoreo et al., 2015). Plasmaization is an extremely sophisticated physicochemical process. In this process, high-energy radicals are formed through dissociation. Although these radicals are electrically neutral. They are unstable and can easily react with other substances to reach a steady state. Electrons are knocked away from the neutral atoms and manipulated by electromagnetic fields. Excitation and relaxation phenomena simultaneously transpire. After an electron collision, the atom remains intact. But absorbs energy to enter an excited state. The valence electrons in it are excited to a higher energy level and then the excited electrons fall back to the normal valence band. Plasma-activated vapor deposition is a non-thermal vaporization process. Under appropriate high-energy ion bombardment, individual atoms escape from the target surface and are deposited at low temperatures due to cascade collisions of atoms (Abegunde et al., 2019).

Plasma control allows effective adjustment of surface porosity and residual stresses to optimize surface properties (Aronov et al., 2008; Li et al., 2022). It also has the advantages of economical, solvent-free treatment, composition control, preparation compliance and sterility. It holds great promise for application in the biomedical field (Cloutier et al., 2015). Organic matter has also been shown to modulate the nucleation and growth kinetics of inorganic matter (Lee et al., 2016). However, existing studies have favored the promotion of adherence growth by biomolecules and structural macromolecules, as well as the inhibition of crystallization by antifreeze proteins (Fang et al., 2011).

Titanium has good biocompatibility (Geetha et al., 2009) and is widely used in biomedical fields. It is usually a mixture of low-temperature hexagonal close packed (HCP) phase and high-temperature body-centered cubic (BCC) phase. The long-term stability of titanium in the biological environment makes the growth of titanium films on polypropylene surfaces an effective technical means of addressing biological infections and rejection reactions. It is crucial to establish a clear mechanistic link between the molecular structure and mechanical properties of the polymer, in particular between energy dissipation hysteresis and dynamic adaptive recovery (Chung et al., 2014). There have been many studies on the static, dynamic and mechanical properties of polypropylene systems. This is generally done through material modification studies, such as blending, grafting and filler methods (Arcana et al., 2007; Watt et al., 2020). Research into large polypropylene surface coating systems, especially with regard to mechanical properties, is still in its infancy. The combination of biostability and controlled thermomechanical properties will therefore open the way for durable biomedical devices (Gentekos et al., 2019; Worch et al., 2020).

The lack of understanding and prediction of the molecular details in the properties for polymer-metal coating interfaces limits the more rational design and application of surface biocoatings. Metal-polymer interfacial mechanisms and their links to mechanisms in biomechanics, including lattice dislocations, surface morphology, nucleation and growth patterns in coatings and energy dissipation theory, need to be further discussed. In this paper, the structure of polypropylene fibers was specifically woven to meet the pull-up properties in different directions. A time-stage modulation strategy was also proposed by profiling the different stages according to the mechanism of nucleation and growth of the polymer surface at the nanoscale. The titanium coating was as well processed by magnetron plasma sputtering technology for satisfactory biocompatibility and mechanical properties *in vivo*. On this basis, the effects of temperature and strain rate on the visco-hyperelasticity mechanical properties of the patch were investigated. The effectiveness and feasibility of the polypropylene hernia patch material for practical clinical applications was also confirmed using uniaxial tensile tests and cytotoxicity tests. The quasi-linear viscoelastic and Gasser-Ogden-Holzapfel (QLV-HGO) constitutive models were further characterized for polypropylene meshes with different coatings. Meanwhile, the kinetic and mechanical properties of the polymers were predicted at the atomic scale. In addition, the interfacial microstructure and mechanisms were investigated and the intrinsic mechanisms affecting the biomechanical properties of the super-viscoelasticity of the patch were explored. The modulation of metal deposition by low-temperature plasma offers a novel way for biomaterials to address biological infections and rejection reactions by effective technological means. Further, it brings great value to the medical industry for medical polypropylene titanium metal nanofilms and implantable biomaterials.

## Results and discussion

### Mechanism of Ti nucleation during vapor deposition on polymer surfaces

**FIGURE 1:**
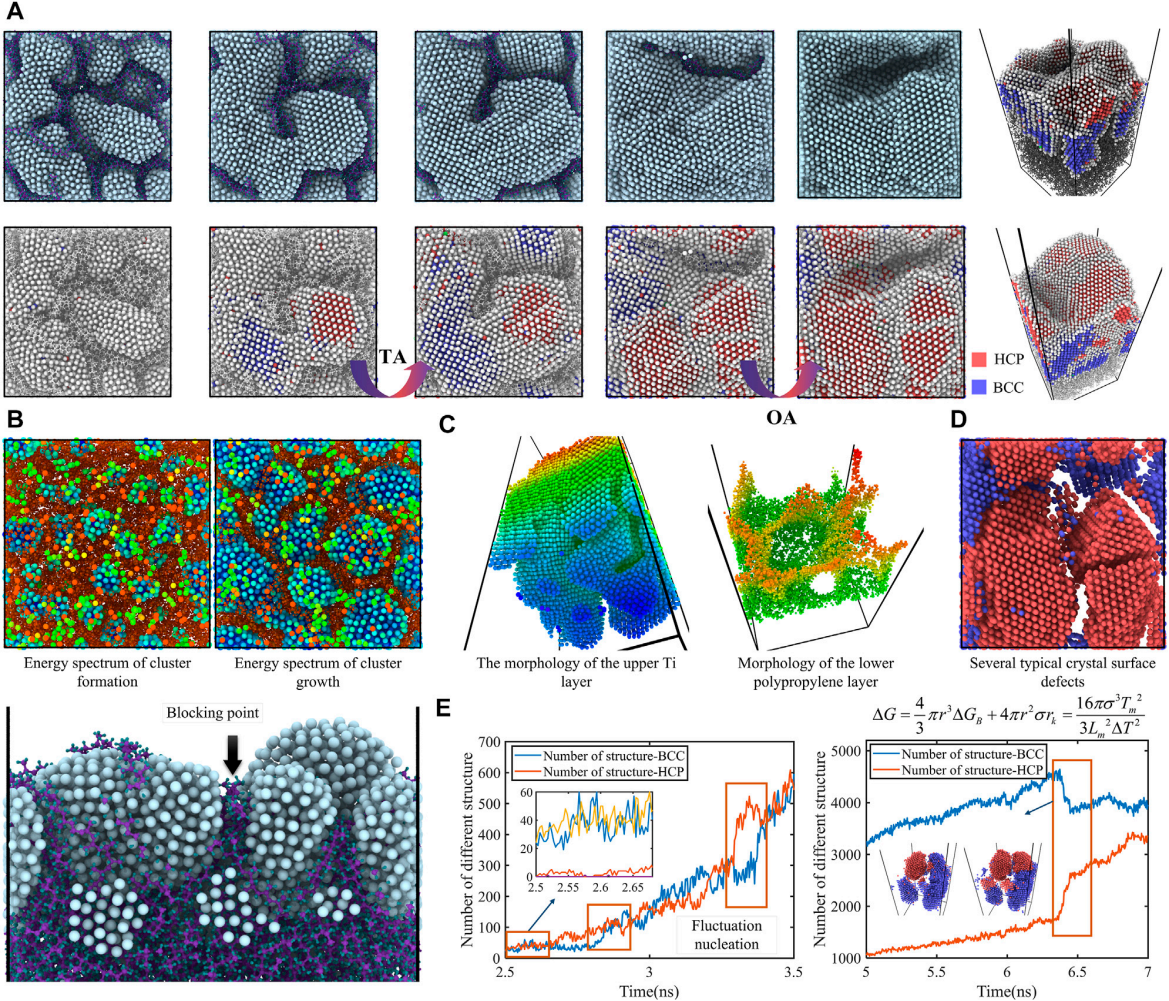
**(A)** Deposition of titanium particles on a polypropylene surface to form clusters based on molecular dynamics simulation. During magnetron plasma sputtering, the incident atoms coalesce on the substrate. From the initial aggregation of numerous clusters on the surface until the formation of complete nuclei, the coating is finally formed. **(B)** The surface potential is distorted when clusters are formed on the substrate after deposition. The titanium atoms moving to the surface are mainly clustered around the relatively low energy clusters, eventually forming stable island structures. **(C)** Stable interfacial structures are formed over a long period of time by deposition. By abstraction of the polypropylene and titanium metal film layers, a bridge-like structure and a garden-like structure are observed, respectively. At this point, a small number of titanium atoms are fully released and embedded in the polypropylene, detailed illustrations of which are shown in Supplementary Figure S2. **(D)** Crystal structure analysis. Defect formation, such as grain boundaries, twinning or dislocation at interfaces. **(E)** The particles in the clusters form nuclei in a fluctuating manner *via* different nucleation pathways. In the subsequent deposition, the formation and interconversion of different crystal structures can be observed.

The surface of the polypropylene patch is relatively rough (Supplementary Figure S1). It was highly susceptible to adhesions to body tissue and irritated the surrounding tissue to produce excessive scar tissue. Patients infected with the disease were required to undergo secondary surgical treatment (Scheidbach et al., 2004). Atomic valence bond analysis, crystal structure, surface roughness reconstruction and grain size largely determine the surface properties. Therefore, it is challenging to adjust the surface properties to obtain coatings with excellent biomechanical and biocompatible properties. The incident atoms formed larger structures through various pathways. As in the surface or sputtering process, this is accompanied by reflection and migration before reaching the substrate, controlled by the magnetron sputtering power and the surface properties of the substrate (Supplementary Figure S2). The nanoclusters are amorphous crystals and the amorphous precursors are generally used as the parent phase for crystallization based on molecular dynamics simulation (Figure 1A). The nanoclusters were relatively stable aggregates comprising tens to hundreds of atoms. The nuclei in nanoclusters were formed in a fluctuating manner through different nucleation pathways. Clusters fluctuating between featureless and semi-ordered states suddenly formed crystals. The nuclei gradually underwent a transition from instability to stability, with the nucleation period obeyed a roughly normal distribution (Nakamuro et al., 2021). Nucleation was a concept of classical nucleation theory, the existence of which has been verified experimentally (Bai et al., 2019). From a thermodynamic perspective, a stable nucleation must have a minimum number of atoms. For droplet nucleation and crystallization, the critical number of nuclei was generally no more than 400 atoms (Lutsko, 2019). This was similar to the findings of this study. A combination of thermodynamically driven size dependence and kinetic constraints on polycrystalline nucleation by potential barriers made the precursor phase a common feature of crystalline systems. Furthermore, when the crystal size was sufficiently small, a continuous structure from the crystalline to the non-crystalline state may exist in that state (Navrotsky, 2011). In contrast to the spatially random nature of nucleation in solution, nucleation on polypropylene surfaces was only present in the nanoclusters formed. In the nucleation period under particle sputtering, the crystal nucleus is in a state of constant formation and dissipation before a stable nucleus is formed (Figure 1E). When the crystal nucleus grows to a certain size, its stability increases dramatically and it does not melt. The size of the stable crystal nucleus is about 1 nm according to molecular dynamics simulations. These nuclei were randomly distributed in a planar pattern on the surface of the substrate. Nuclei continued to grow, but did not increase significantly. The particles after collision and migration grew attached in the middle of different crystal structures, forming amorphous nanoparticles. Further, they combined with different nuclei, resulting in the growth of particles in amorphous clusters, climbing from the amorphous parent phase to the nucleus (growth of transitional appendages, TA). As the nuclei became fused and stabilized, there is an oriented attachment (OA) growth pathway through individual particles or clusters on the crystalline surface (Figure 1A). During the deposition process, irregular amorphous crystals reflected or absorbed the impact atoms beyond the nucleation site. There was then a gradual transition to a regular state, preferentially forming another complete atomic layer on the surface of the original nucleus (Figure 1A).

Amorphous crystals on the surface of HCP might form nanoscale chemically ordered superstructures and transition to the BCC phase. The energy barrier to their transition was almost zero and could be overcome by atomic vibrations (Fu et al., 2022). The second pathway of CPA, which is carried out by repeated OA to specific lattice-matched crystalline facets, tends to occur in the mid-to late-stage of crystallization. Both nucleation pathways resulted in rapid changes in geometry and crystalline phase. At the same time, the interfacial free energy had a strong influence on the CPA pathway due to the size of the free energy barrier affecting it. The contribution of free energy often led to structural stability depending on the size of the crystal (Navrotsky, 2011). In order to generate single crystals in a polymer system, the joining event must accommodate the structural differences between the two phases (Li et al., 2013). Either a structural match was made at the interface or a phase transition took place after the formation of amorphous crystals. However, the growth of metals on polymers was difficult to adapt to the lattice of crystals and crystals through chain entanglement. Its preference was for fixed-point lattice matching of particles, nanoclusters and crystals. If the nuclei were interconnected and their crystallographic orientation was hardly to deflect into a perfectly parallel arrangement by the action of the polymer, defects such as grain boundaries, twins or dislocations would form at the interface (Banfield, 1998). As a result, particle attachment crystallization continued as the nuclei gradually fused with each other and completely covered the polypropylene molecular chains. Further, the film morphology was formed by the attachment of crystalline arrangements of particles on lattice matching surfaces. Alternatively, it crosses twin boundaries and stacking faults (Penn and Banfield, 1998) that are distributed with these irregular defects (Figure 1D).

CPA was influenced by the surface potential energy of the different substrates and the incident energy of the Ti particles. When the atoms on the substrate surface were uniformly distributed, the surface potential was essentially the same as the substrate surface morphology. However, when agglomerates were formed on the substrate after deposition, the surface potential around the agglomerates was distorted. The rough surface and non-equilibrium nanoclusters affected the particle-particle interactions prior to adsorption. Diffusion-dominated nucleation was replaced by potential-energy-dominated nucleation, resulting in different single-crystal structures and microstructures. The trajectory of the incident particles was deflected toward the clusters. The surface potential energy is absorbed by the low potential energy islands on either side at the end of the collision with the molecular chain (Figure 1B). The high energy region (blocking point) between the two clusters also shielded the incident particles. The surface roughness and crystal structure of the film layer was not static and would change with environment and time. The molecular chains were embedded in the crystal structure as well as the non-uniform surface structure of the crystal and the surface charge distribution trigger the layered HCP and BCC structures. This was dependent on symmetry of the potential energy and forces. Figure 1C shows the change in the initial structure of the lower polypropylene layer and the upper Ti layer at the end of deposition. Due to the low intermolecular forces between the polymer chains, there was a large drift of the molecular chains during deposition and the nucleation islands grew. At the same time, the empowerment of the molecular chains extended upwards, preventing the formation of grain boundaries, which resulted in the formation of a unique nursery ring structure in the lower polypropylene layer. Under the influence of the polypropylene, bridges appeared in the upper titanium layers, which were special structures formed by the high-energy molecular chains of polypropylene crossing them during deposition. Appropriate tuning would enhance the bonding of titanium at the atomic scale, increase the interfacial strength and prevent the metal coating from flaking off. However, unsuitable deposition process parameters could result in the formation of micropores in the deposited films and a decrease in film density. As deposition proceeds, the eventual formation of a multi-surface structure with the growth of particles attached directionally to lattice-matching sites was studied visually. For multi-segmented structures, the rates of attachment and detachment were dependent on the density of kink sites and the energy required to generate new kinks (De Yoreo et al., 2015). It was surprising that there was an interconversion of the BCC and HCP structures during the gradual deposition process (Figure 1E). The transformation from HCP to BCC structure was an additional plastic deformation mechanism comparable to twinning for metals and alloys with HCP structures (Wu et al., 2016). There was an important difference in the polymer systems as the film layers were cut by molecular chains and competition between monomer growth and growth of adherent particles of different sizes had to be considered. As the average thickness of the film increased further, the mesh developed into a continuous film. At this point the incident atoms started to hit the same kind of atoms, reflections or desorption were significantly reduced due to the change in binding energy and the multi-system problem evolved into a monometallic system.

Classical nucleation theory suggested that the free energy of the volume accounted for two-thirds the work of nucleation, with the remainder was provided through thermodynamic energy fluctuations, which was the nucleation barrier. The order of magnitude associated with the free energy barrier to nucleation compared to the size of the thermal energy was a key factor in determining the number and nature of the particles produced. Unlike the assumptions of classical nucleation theory, the surface and free energy of non-classical nucleation were not constant for particle size. The nucleation potential barrier was situated between the two extremes of the high-energy barrier and the no-energy barrier. The existence of intermediate structures with lower surface energy or volume free energies provided an alternative pathway of circumventing the high-energy barrier of homogeneous nucleation (Wang et al., 2014). During the transition process, the presence of a series for local intermediate states with minimal energy led to the existence of a series for sub-stable phases. Due to thermodynamic energy fluctuations, these sub-stable phases were continuously replaced by more stable phases. During the relaxation process, a large particle was formed and thus located at the low point of the free energy surface. In contrast to conventional nucleation, nucleation of accreting atoms in vapor deposition by this process was not affected by subcooling or supersaturation, but only by occasional collision and agglomeration events of accreting atoms. The growth started with agglomerative transformation, direct collisions and binding with other particles. After nucleation, it shifted to a growth mode that absorbs proliferating atoms.

### Time-dependent regulatory mechanisms based on polymer chain entanglement

In this section, a time-dependent power spectrum was proposed and completed with a dual regulation of biomechanics and biocompatibility. The deposition morphology and analysis at different parameters are shown in Supplementary Figure S4 and Supplementary Figure S5. It can be concluded that the surface varied considerably at different stages while showing a regular trend of evolution. However, the analysis of cytocompatibility and biomechanics required to be further quantified by constraint planning. This would avoid generating singular values or getting stuck in local optimization and ultimately give accurate results. The surface is extracted and the parameters derived (Supplementary Figure S6), and the arithmetic mean deviation of the profile and the maximum height difference of the contours were calculated.

**FIGURE 2:**
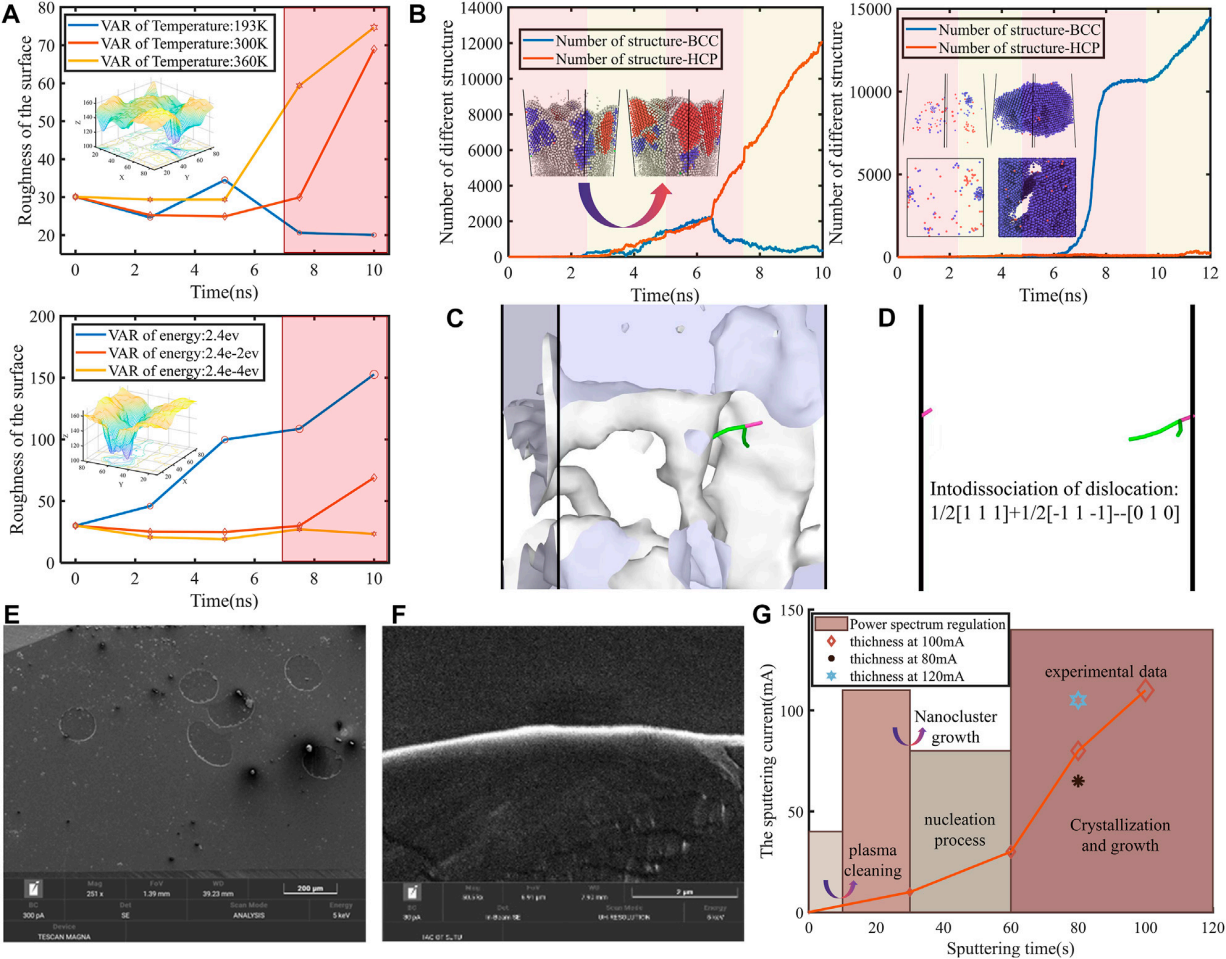
**(A)** Surface properties based on substrate temperature and sputtering energy. Higher energies lead to higher surface roughness and the pink area is the stage where a large number of nuclei grow. Large areas of polymer are covered and this stage is a critical period for the reaction mechanism. **(B)** The different undertones represent different periods of sputtering, with relaxation occurring at the end of each period. The free energy determines the thermodynamic preference. It is up to diffusion and particle relaxation to determine whether thermodynamic processes occur. The second picture shows the formation of massive individual crystals by suppressing nucleation until explosive nucleation occurs when the thermodynamic driving force becomes large. **(C)** Schematic diagram of dislocation analysis. Within a certain range of dislocation density, the plasticity of the material increases with more dislocations or twins, that is, it can withstand higher external loads and has better ductility. **(D)** The movements of dislocations, the green line represents the dislocations with Burgers vector [111] and magnet line represents the dislocation with Burgers vector (001). **(E)** Analysis of the cross-section of the encapsulated polypropylene and the thickness of the titanium coating. Schematic showing the variation with growth for the thin film layer, the circular polypropylene fibers were embedded in paraffin wax and frozen sliced. **(F)** A partial magnification of the Ti layer is used to accurately measure the thickness. **(G)** The thickness of the film growth is used to predict the cycle time in sputtering, then adjustments are made. The thickness variation of the film layer is also verified experimentally for different sputtering parameters.

When the incident energy of the Ti particles increased, the surface roughness first decreased during the deposition process due to the relatively rough structure of the initial polypropylene. Whereas the particle filling in the voids resulted in a denser surface, demonstrated in Figure 2A. The formation of porous defects was less likely as the temperature of the substrate increased, due to the higher energy titanium particles crossing the energy potential barrier more easily. The deposition of titanium then gradually developed from the filling of clusters in the voids to the growth of agglomerates and crystal nuclei. Roughness increased with power as the fusion of grain boundaries was impeded by the long chains of the polymer that gave high energy and formed grooves. The nucleus grew further until the polymer was completely encapsulated and gradually separated from the polymer. During film formation on non-polymer surfaces, the roughness tended to become progressively lower as the sputtering power increased (Chen et al., 2015). The overall BCC structure appeared to dominate as the sputtering power decreased. The HCP prevailed only when the sputtering power was highest, and details of the crystal structure evolution are shown in Supplementary Figure S7. The two nanoparticles were very close to each other before the collision. They had strong interactions with each other, which facilitated the alignment of their crystal orientations. The strength of this interaction was powerful enough to induce rotational and translational motions that aligned the nanoparticles prior to collision (Bronstein et al., 2012). Upon completion of alignment, the nanoparticles immediately merged by attractive forces. The higher sputtering power therefore brought about rapid crystal formation. The degree of molecular chain embedding increased gradually as the substrate temperature increased. It can be observed that the effect of temperature on the lattice was comparatively obvious. As the substrate temperature elevated, the Ti atoms were more likely to form regular BCC structures during the deposition process. Moreover, the formation was more advanced, while the proportion of BCC gradually increased. BCC and HCP nucleated competitively, with HCP finally occupying the steady state due to thermodynamic relaxation of the internal particles (Figure 2B). The free energy landscape determined the thermodynamically preferred shape of the cluster structure. Further, the shape and size distribution were dictated by the diffusion of clusters and particles as well as the relaxation within the particles as to whether thermodynamic processes occurred or whether this free energy minimum could be reached by crossing the potential barrier. The BCC structure offered better plasticity compared to the HCP structure. It can be demonstrated by analyzing the dislocation density inside the film layers at different incident atomic energies that the dislocation density first increased and then decreased with rising incident atomic energy levels. When the incident energy was too low, the crystal structure was less affected and the internal slip was not sufficient. However, in contrast, when the incident energy was too high, the slip in the crystal structure was excessive and dislocations moved to the boundary of the structure and disappeared (Figures 2C, D).

By calculating the thickness of the film growth, the sputtering period could be predicted, including the clumping period, the clumping growth and fusion period, the nucleation period and the growth period (Figure 2G). This was done until the crystallization period when the surface was completely covered, thus completing the adjustment of parameters. It was possible to visualize the thickness of the circular sample of polypropylene fibers after freeze sectioning as shown in Figure 2E. The frozen slice of the fibers is enlarged to obtain a microscopic diagram of the Ti coating, as shown in the white bright band in Figure 2F, which allows accurate calculation of layer thickness. The current stage of nucleation and growth could be deduced by analyzing the thickness of the titanium coating. The first stage of cluster formation required low energy. This was to avoid greater damage to the substrate, while achieving a plasma clean surface and removing trace gases present in the adsorption (Song et al., 2014). The second stage, cluster growth and fusion, demanded higher power. This was for the polypropylene molecular chains to passively acquire the ability to extend upward, thus preventing the formation of a homogeneous film at the interface. Since materials with different coefficients of thermal expansion as well as tensile strain rates, the effect of trace deformation *in vivo* on the mechanical properties of the film layer should be avoided. Simultaneously, the initial stresses during film formation should be reduced. In the third and most important stage of nucleation and growth, mechanical properties were characterized by analyzing the changes in crystal structure, the formation and evolution of defects within the crystal during deposition. The HCP structure of metallic titanium at room temperature had many slip systems, which were prone to dislocation and twinning defects under the action of external forces. The presence of these defects hindered the nucleation of subsequent dislocations and twins. Therefore, there was a preference for building BCC surfaces that can withstand higher external loads and have better ductility. During the deposition process of Ti atoms, collisions between the incident and deposited atoms continuously occurred. The formation of dislocations or twins by sliding was based on the development of a certain stable lattice structure, where collisions of incident atoms caused atomic movements inside the lattice. Analysis of the deposited stable structures showed that no dislocations were formed at temperatures too high or too low. However, dislocations were present at temperatures of 273 and 300 K, but with different types of dislocations. The deposition processes at 193 and 360 K were analyzed, in which dislocations were generated and subsequently annihilated, resulting in a final structure without dislocations. The mechanism of dislocation generation was that dislocations moved separately along different new slip surfaces in the crystal structure, continuously moving towards the boundaries and finally annihilating. The dislocation decomposition was 1/2 (1 1 1) + 1/2 (−1 1–1)–(0 1 0). The subsequent dislocation movement to the boundary was restricted and the dislocation lines became shorter. This dislocation reaction decreases the energy of total structure, resulting in a more stable state around the place where this reaction occurs. It is noticed that (001) edge dislocation is observed in experiments, which is stable under some temperatures. Here, the atoms around (001) edge dislocation undergo greater stresses than other atoms, facilitating their movements during the deposition. This can be demonstrated by the molecular simulations that the (001) edge dislocation moves farther and more quickly than other dislocation types. Finally, they move to the boundary, and vanish to ensure the structure consisted of deposition atoms in a stable state. The very thin Ti film layer formed by the deposition had good strength on the surface and would not fail. At this point an appropriate power reduction was required to modulate the lattice and form the BCC structure. As the deposition continued, the nuclei gradually fused with each other. The polypropylene molecular chains were also completely covered by the accumulation of particles spanning twin boundaries and stacking faults. As the strengthening of the material was mainly based on minimal lattice mismatch (Jiang et al., 2017) (Figure 2B), it formed large scale structurally perfect single crystals by suppressing nucleation until explosive nucleation occurred when the thermodynamic driving force became large. The lower layer was completed with a drifting island structure, while the upper layer was a homogeneous BCC single crystal that was regulated by a time-sharing regulation strategy. This facilitated direct contact with the cells and the climbing of the cells. It also afforded better ductility to avoid damage to the structure.

### Visco-hyperelasticity mechanical properties in the transient critical state

The weave structure and mesh aperture size had a significant impact on the mechanical properties. Currently, patches were available in three types of pore size, and the appropriate pore size needed to be selected for the patient. The mesh patch was created by weaving polypropylene fibers arranged in a rhombic structure. The weave structure strongly influences the performance of the mesh patch (Supplementary Figure S8). In this paper, a second weaving process was used, which was more conducive to the mesh conditioning and anisotropy analysis. The monofilaments had a diameter of approximately 120 μm and consisted of many rhombic lattices with an angle of 34.7°. Experiments were carried out by depositing metallic titanium on the surface of the polypropylene for stretching in order to investigate the effect of different surface properties on the stretching.

**FIGURE 3:**
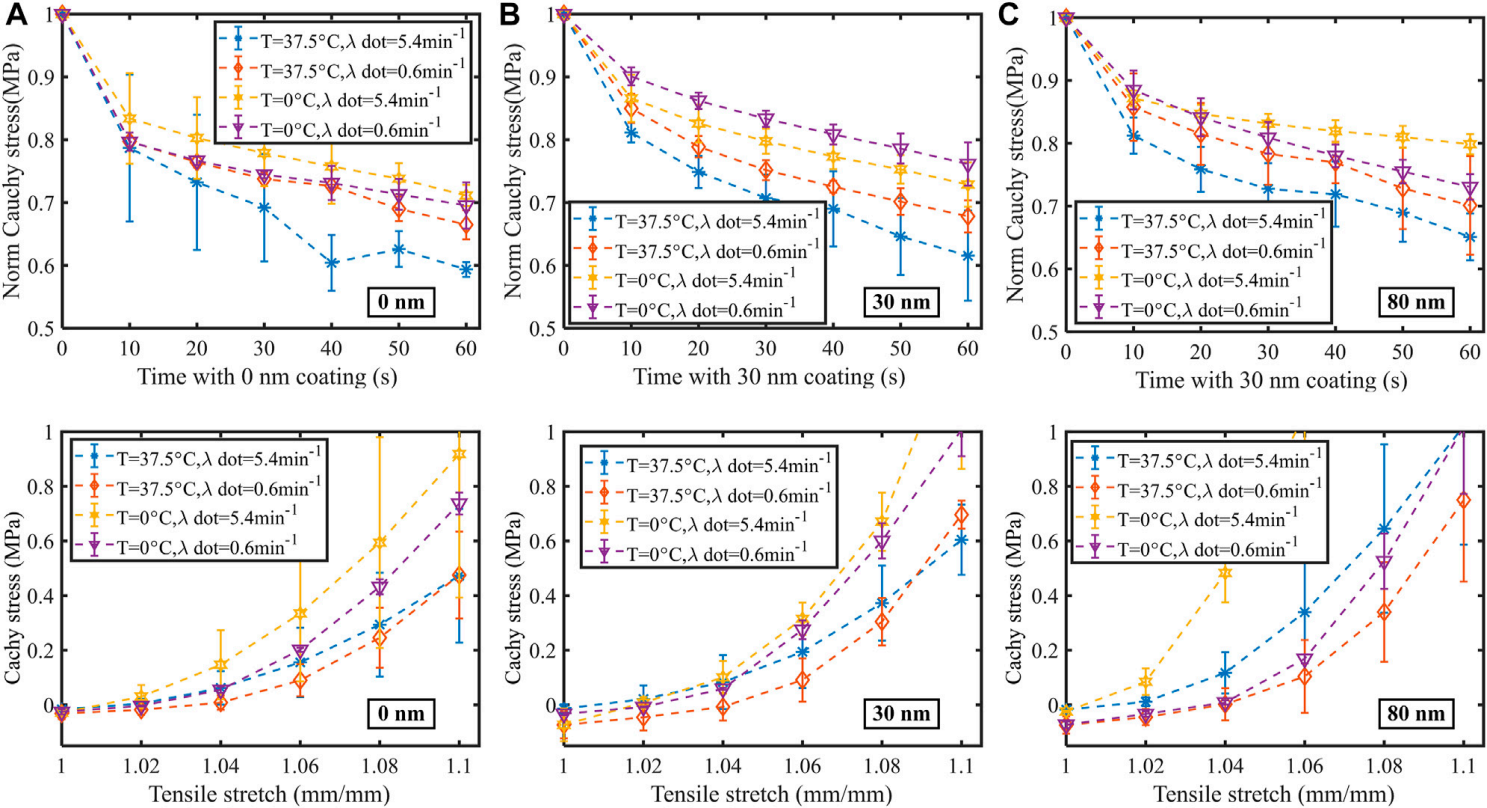
Stress relaxation and uniaxial tensile mechanical data of polypropylene patch coated with 0, 30, and 80 nm thickness of titanium are provided out through **(A–C)**. The effect of two ambient temperatures (0°C and 37.5°C) and two loading strain rates (6 and 54 mm/min) on changing the visco-hyperelasticity properties of the strips is investigated. All plots show how the mechanical properties of the strips vary with the different coatings. The first row shows the measured data for strip relaxation. Meanwhile, the second row shows the measured data for strip stretching. Each row shows a different coating thickness. The data are shown as dashed lines with error bars, indicating the visco-hyperelasticity mechanical properties under the respective conditions. The data curves fully compare the effect of temperature and strain rate on the visco-hyperelasticity of the strips.

Numerous experimental studies demonstrated that polypropylene patches exhibited purely elastic properties over a deformation range of 0%–10% of the engineering strain (Lubowiecka et al., 2022). This range of elongation basically covered the elastic deformation process of the implant *in vivo*. It supported the view that the implant did not massively damage and exercise the deformation of the human abdominal wall. In macroscopic tensile mechanical testing, elongation was controlled at 1.0–1.1. Comparative analysis of the visco-hyperelasticity mechanical response generated by strip stretching was generally used as a routine method (Podwojewski et al., 2014). The experimental results indicated, that the thicker the thickness of the coating, the weaker the bond strength and the higher the elastic stiffness of the patch material. Coating of the patch by magnetron sputtering can trigger changes in the microstructure of the material, leading to modifications in the macroscopic properties of the material. The coating treatment not only improved the biocompatibility of the material, but also enhanced its mechanical properties, such as toughness, to a certain extent. In patients with more severe lateral abdominal wall leaks, the absence of abdominal tissue can lead to a severe reduction in biomechanical properties. As a result, such patients often required higher performance hernia patches for their treatment. Therefore, in the clinical management of certain severe hernia leaks, coated hernia patches are the best solution to take advantage of their superior mechanical properties (Figure 3).

The influence of temperature and strain rate on the mechanical properties of the strips could also be observed in some figures. Stress relaxation and uniaxial tensile mechanical data of polypropylene patch coated with 0, 30, and 80 nm thickness of titanium are provided out through Figures 3A–C, respectively. For each type of coated patch, we conducted a centralized comparative analysis of stress relaxation and uniaxial tensile mechanical data measured under two different tensile strain rates (0.6 and 5.4 min-1) and two different temperature environmental conditions (0°C and 37.5°C) based on the research idea of controlling variables (a total of four groups of data were listed in figure for each type of mechanical experiment). Just as shown in Figure 3, each group of characteristic data is extracted and displayed in the form of dotted lines, where the stress relaxation experimental data are listed in above legends, and the uniaxial tensile mechanical data are listed in below legends. From the viscous relaxation function theory combined with the time-coursed normalized Cauchy stress data detailed in the stress relaxation legend, it could be seen that the faster the normalized Cauchy stress decreases over time, the greater the viscosity the material exhibits. Similarly, combined with the hyperelastic tensile Cauchy stress-stretch data detailed in uniaxial tensile legend, it could be seen that the higher the Cauchy stress data as the stretch increases, the greater the stiffness the material exhibits.

By comparing and analyzing the mechanical properties of a certain type of plated thickness patch strip with mechanical test data, it is not difficult to find that our results echo the following conclusions: the greater the tensile strain rate, the more significant the viscosity and stiffness of the material; The higher the ambient temperature, the stronger the viscosity and lower the stiffness of the polypropylene material. At the same time, we looked at the differences in visco-hyperelastic mechanical properties of different coated thicknesses and found that the higher the coating thickness, the less significant the adhesive properties of polypropylene materials, while the more significant the stiffness properties. The higher the temperature, the lower the tensile stiffness of the strip and the more viscous the stress relaxation behaves, while the reverse was also true. This mechanical phenomenon was the same as the familiar laws of physics. From a microscopic perspective, the higher the temperature, the more violently the molecules and atoms within the object vibrate and the weaker the forces between them. For polypropylene patches at increasing temperatures, they tended to exhibit loosening and slipping of the fiber bundles, which undoubtedly resulted in macroscopically low stiffness and high adhesion. In this paper, values were given for the mechanical properties of hyperelastic patches with different degrees of coating at different temperatures, as described in the Supplementary Material S1. It is easy to realize that the thicker the coating, the stronger the constraints on atomic and molecular vibrations at the microscopic level. The results from this section could give some qualitative/quantitative evaluation criteria for good or bad results of abdominal wall surgery in patients after hernia repair surgery across different environments and seasons (Figure 3). The higher the strain rate, the higher the tensile stiffness of the strip and the stronger the stress relaxation viscous properties, and the opposite held true. This phenomenon reflected the mechanical properties manifested by viscoelastic materials. From a microscopic point of view, the macroscopic profile of the polypropylene strip changed continuously during the stress relaxation process. At the same time, its internal molecular slippage was accompanied by some reversible bond breaking and exchange. Relatively rapid readjustment of entangled molecular chains was involved. When the elastomer was deformed at very low rates, the loading time was long enough to cause unrestricted readjustment. As the rate of loading increased, the relaxation time decreased, and the amount of internal coiling, curling and kinking changed. When the elastomer was deformed at very high rate, the readjustment of the internal structure was limited, and the relaxation mechanism did not occur. The rate sensitivity laws for modulus and strength of viscoelastic materials explained the properties of strip super-viscoelasticity. The computational results of the model presented in this paper and a description of additional experiments at low temperatures can be found in the Supplementary Material S1. Similar to the previous animal experiments (Deeken and Lake, 2017), the results in this section illustrated that the mechanical state of the 30 nm patch implanted in the abdominal wall fully met the daily physiological demands of patients in different states of physical activity after abdominal wall surgery. Temperature sensitivity and strain rate sensitivity were also further revealed.

### Analysis of microscopic deformation mechanism

**FIGURE 4:**
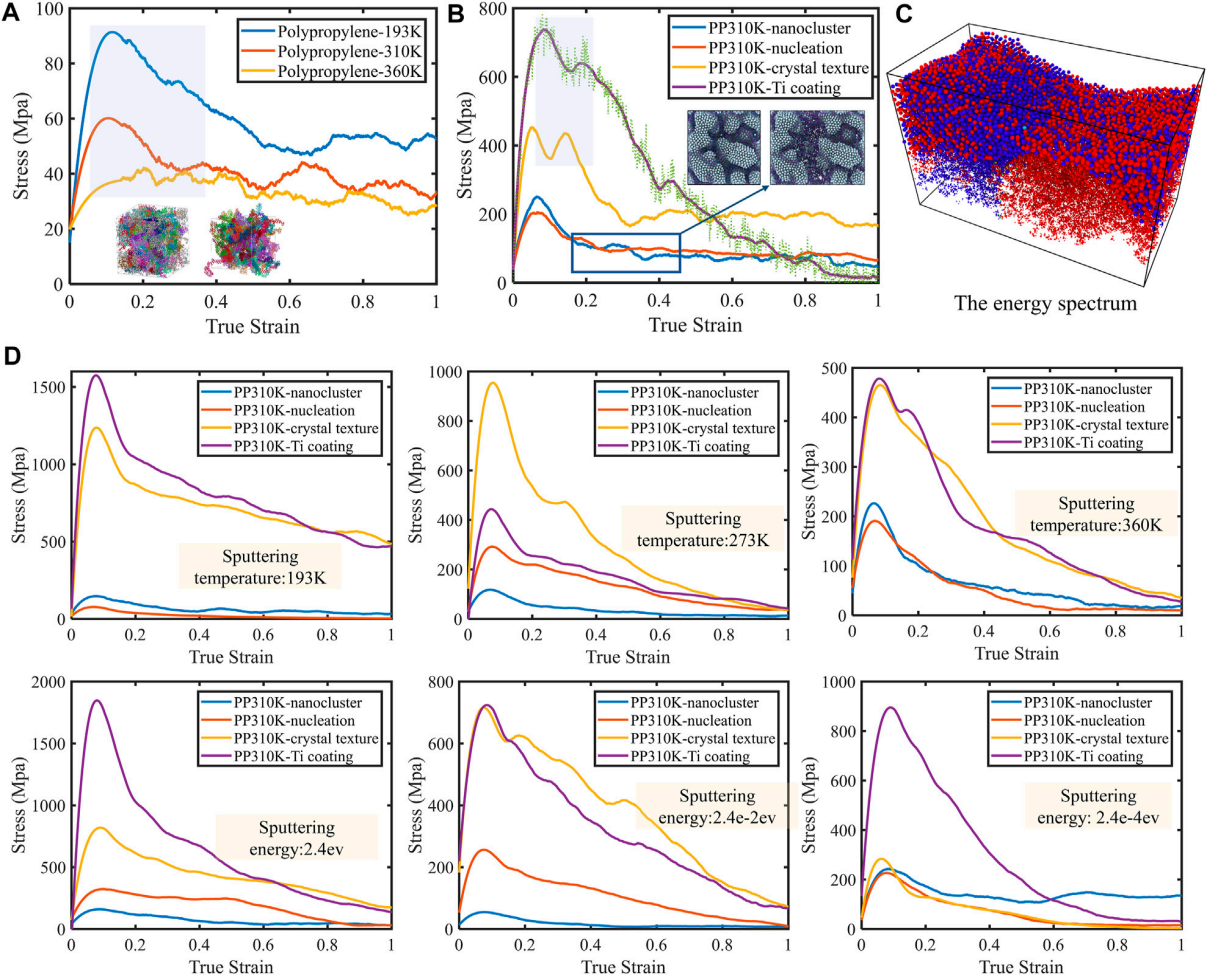
**(A)** The effect of temperature on the stress-strain behavior. **(B)** Characteristic evolution of the interface during stretching. When no homogeneous membrane-like metal film is formed, there is essentially no change in the agglomerates during stretching, when the movement of the molecular chains is predominant. **(C)** Schematic diagram of the change in particle displacement during stretching. As the displacement increases, the color gradually changes from blue to red, indicating a significant change in the position of the lower polypropylene layer. The longer polypropylene chains undergo great deformation, which involves a mechanism of decoupling. **(D)** The effect of different surface roughness and crystal structures as well as different periods on the mechanical properties.

As shown in Figure 4A, the deformation of the material increased significantly under the action of an external force, with the deformation remaining relatively stable over certain subsequent intervals. Molecular dynamics simulations were used to investigate the deformation mechanisms during uniaxial tensile deformation of amorphous polypropylene polymers and to explore the effect of temperature on the stress-strain behavior. It was well known that the stiffness of the material decreased with increasing temperature, which was consistent with the experimental results. Figure 4B shows the interfacial properties and the tensile evolution mechanism. The different periods of evolution (cluster formation, growth and fusion processes, nucleation and growth, film formation) had a strong influence on the stress-strain. The microstructure also revealed that the morphology of the surface clusters remained unchanged when the patch was stretched as the unfused nanocluster structure was formed. The underlying polypropylene underwent chain-to-chain slippage and chain-to-metal slippage, resulting in surface nanoclusters resembling continents in the movement of small plates. Although the overall shape remained the same, it would drift. This conclusion was verified (Figure 4C). This was because of the ability of the polypropylene molecular chains to entangle with the Ti coating. Forming the above-mentioned bridge-like links and increasing the strength of the interface. In the case of changes in particle displacement during stretching, a significant shift in the position of the lower polypropylene layer can be seen. At the same time, the long polypropylene chains underwent considerable deformation. If the biocompatible properties of Ti nanoclusters can be well realized, the design will completely change the scenario for Ti applications. Temperature effects and stress effects in the interface can also be addressed. The crystallinity of the polypropylene did not change significantly during Ti deposition and microstretching (Jia et al., 2015). In all cases, as strain increased, the alignment of the chains in the direction of loading increased and the crystallinity rose. The relationship between the tensile curves and the surface properties and crystal structure was further analyzed (Figure 4D). A distinctive feature was the gradual fusion with the third stage nuclei until the formation of the membranous structure, when the mechanical properties underwent a step change. That is, the two curves in the upper zone were clearly separated from the two curves in the lower zone. The small change in mechanical properties during the formation of the nanoclusters demonstrated the discrete effects mentioned earlier, which led to more flexible properties. As the sputtering temperature increased, the strength of the interface obtained gradually decreased. This was due to the gradual shift from the HCP structure to the BCC structure as the temperature increased. In addition, when the surface was extremely flat, the strength was more uniform. Also, this heightened the interfacial effect as there is no good tensile fracture point. Meanwhile, sputtering power can have a large effect. The same sample prepared at high power had a great value of strength when HCP dominated. As the power was reduced, the intensity of the sample decreased when the BCC structure became dominant. The last graph shows particularly clearly the jump in intensity from stage 3 to stage 4. This was due to the explosive growth of the BCC structure mentioned already. Weak interactions were found within and between polypropylene chain molecules in the microporous regions formed within the polymer matrix region due to the strong load transfer capability of the interface.

As a result, it tended to propagate through the interface while forming a more homogeneous film layer stretch (Supplementary Figure S9). With good ductility, a homogeneous film-like metal layer would form if a large number of dislocations were not formed. The presence of long chains in the interface between polypropylene and Ti also limited the propagation of damage. The high degree of bridging entanglement led to a high load-bearing capacity and the interface had a higher interfacial strength. Furthermore, the load was transferred to the interface by this bridging action. Non-adhesive interactions between chains can play a key role in the elasticity, yielding and softening processes of materials. Continuous tensile deformation could cause significant changes in the entanglement of bridging polymer chains. The results of the energy distribution revealed that the elastic and yielding regions were mainly controlled by non-adhesive interactions between chains. That is, the van der Waals forces between the polymer chains increased, while the strain hardening region was controlled mainly by dihedral motion within the chains (Hossain et al., 2010). Based on the total energy, the influence of the titanium layer on the various components of the energy was explored. In the elastic and strain softening regions, the non-bonding energy increased sharply, which was associated with the chain slip mechanism. In the presence of metal coatings, strain softening and stress softening gradually became less pronounced due to the perturbation of non-bonding (van der Waals force) interactions between the chains during this process. Instead, chain-to-chain untwisting occurred in this region. Notably, the non-linear mechanical response of the visco-hyperelastic intrinsic model polypropylene network was closely related to its degree and rate of deformation. At higher temperatures, irreversible strain or stored potential energy of the macromolecular chains was released, leading to dimensional changes and film shrinkage (Supplementary Figure S10). The energy changes in bond lengths and bond angles were minimal compared to the changes in non-bond energies.

### Biological validation

**FIGURE 5:**
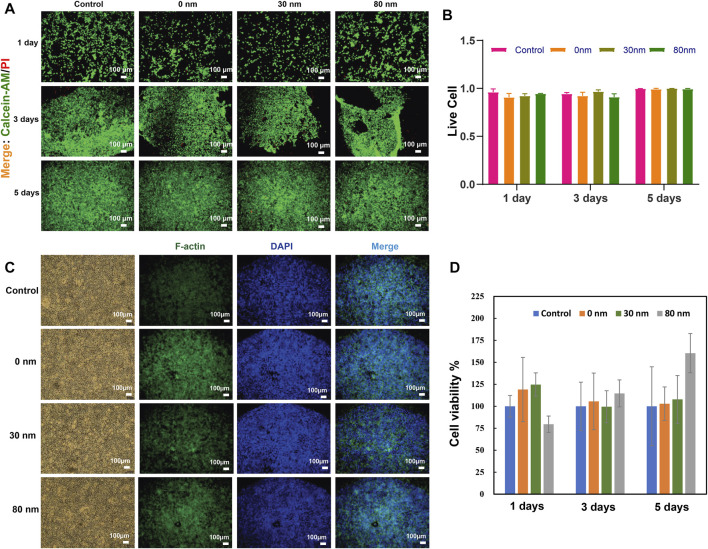
*In vitro* biological validation with L929 cells. Imaging **(A)** and counting **(B)** of live/dead cell assay using the Calcein-AM/PI double staining kit. Cytoskeleton assay with FITC-Phalloidin at day 5 **(C)**. Cell viability/toxicity assay with the CCK8 kit **(D)**. Different mesh samples (0, 30, and 80 nm) were co-cultured with L929 cells for different periods of time (1, 3, and 5 days), respectively. Each bar represents the mean ± standard deviation of three biological replicates. There is no significant differences between the groups according to the *t*-test.

Biological validation is a fundamental and necessary test to assess the biosafety and biocompatibility of biomaterials. The meshes (0, 30 and 80 nm) were co-cultured with L929 cells and J774A.1 cells for different times (1, 3 and 5 days), respectively, to investigate the biosafety and biocompatibility of the different meshes. Various assays were applied to evaluate biosafety and biocompatibility. The results of live/dead cell staining showed that the live cell rate exceeded 90% for all periods (Figures 5A, B and Supplementary Figure S11A,B). In addition, the cytoskeleton of fibroblasts was stained by FITC-Phalloidin immunofluorescence staining. The results revealed that the cells were morphologically and structurally intact and grew well at different incubation times and in different patch extracts (Figure 5C, Supplementary Figure S11C, Supplementary Figure S12–S15). The results of the CCK-8 test (Figure 5D and Supplementary Figure S11D) showed that both L929 and J774A.1 cell exhibited normal growth and cell viability under co-culture at different times. It indicated that all meshes were free of cell viability. All results were not significantly different between the groups. In summary, these results demonstrated that good biosafety and biocompatibility were achieved for both the mesh substrate (0 nm) and the different modified meshes (30 and 80 nm).

Currently, mesh transplantation is a common method of treating hernias. However, studies showed that the available mesh still had some shortcomings. Long-term implantation in the body could cause a number of syndromes that reduced the benefit to the patients (Kokotovic et al., 2016). It was therefore a challenge to design and develop meshes that would maintain good biological function in the patient’s body over time. Biomaterials played an important role in medical applications and their surface properties determined their performance and biocompatibility (Hou et al., 2020). The meshes designed in this study were biologically verified and were proven to have excellent biosafety and compatibility. The biosafety of the different meshes was evaluated in diverse ways in 2 cell types (mouse fibroblasts cell line and mouse macrophages) cultured *in vitro* using different assays (Figure 5, and Supplementary Figure S11–S15). The results showed that all the meshes were not cytotoxic. After different co-culture times, the cells had normal morphology and structure and good growth activity. Based on cytoskeleton staining and microscopic observation of cell morphology, it confirmed that the meshes did not affect cell morphology. At present, the CCK8 method is a preferred method for detecting cell viability/cytotoxicity with high a high degree of accuracy and reliability (Stoddart, 2011). In this study, CCK8 was used to detect the effect of meshes on the cells. Intriguingly, after a long period of co-culture (5 days), the cell viability of L929 was slightly higher than that of the control. In order to realistically simulate the response of a foreign body *in vivo*, a macrophage cell line (J774A.1) was chosen to carry out the experiments. The results showed that the survival rate of macrophages was more than 95% at both 1 day and 3 days. After a longer period of incubation, the cell survival rate decreased, but remained above 90%. This was probably due to the contact inhibition of cells (Supplementary Figure S11). Macrophages were involved in the process of inflammatory response.

## Conclusion

In this paper, the regulation of residual stress, grain size and surface morphology was accomplished by plasma control. Moreover, the effects of the structure and dislocations of HCP and BCC were analysed. The nucleation mechanism of the polymer substrate was revealed for the first time, and the regulatory mechanism of the polymer-titanium metal interface over time was proposed. The unique strain-rate sensitivity and temperature dependence of the prepared polypropylene patch materials were investigated from several perspectives. The apparently superior biomechanical properties were developed with contributions from atomic valence bonding analysis, crystal structure, surface roughness reconstruction and grain size. Precursor phases in the kinetic constraints of polycrystalline nuclei were a relatively common feature of crystalline systems based on thermodynamically driven size dependence and potential barriers. Amorphous crystals formed under the perturbation of long polymer chains contained different nuclei. Further, nuclei in amorphous clusters grew by climbing up from the amorphous parent phase and then passed through a directionally attached growth path of single particles or clusters. Nuclei can also be grown through the crystalline path of cluster attachment. However, polymer-bound nanoclusters were difficult to repeatedly attach to a specific lattice-matched crystal surfaces due to defect formation. Molecular dynamics simulations were used to explore the main deformation mechanism of polypropylene polymers under uniaxial tensile loading. Different strain rates and temperatures were experimented and simulated to investigate the effect of these external factors on the mechanical properties of the polymer. The mechanisms of intrinsic deformation in elastic, yielding and strain-hardening regions were elucidated using an intrinsic constitutive model. The tensile stress-strain of the patch growth state at different film thicknesses and temperatures was simulated by molecular dynamics. Meanwhile, the intrinsic mechanisms of free volume and entanglement density as well as coating adhesion are investigated. The thicker the coating, the stronger the constraints on atomic and molecular vibrations at the microscopic level, bringing high stiffness and low adhesion at the macroscopic level. As the loading rate increased, the relaxation time gradually decreased, and the amount of internal coiling, curling and kinking changed. These explained the super-viscoelastic properties of the strip. The titanized polypropylene patch material presented in this paper had excellent biomechanical properties. It was modulated by metal deposition in low-temperature plasma, linking the stress-strain response to the deformation mechanisms responsible for the mechanical behaviour from multiple scales. This opened a new path for biomimetic materials, thus facilitating the development of patch network preparation structures with good biological stress properties. Furthermore, this study explained its intrinsic mechanism, which to certain extent led to a shortening of the complete development cycle of alternative biological tissue products. The mesh showed good biosafety and biocompatibility in combination with these biologically validated test data. The mesh shows good biosafety and biocompatibility. As the ultimate goal of the designed mesh is to be implanted in patients for the treatment of hernias, further animal experiments are required to validate it and to conduct long-term *in vivo* testing and observation. Overall, this study accomplished the dual modulation of woven structure and coating to meet the mechanical properties and biological requirements of the human body, bringing great value to the bio-implant medical industry.

## Materials and methods

### Cell culture

L929 cells (kindly provided by Cell Bank/Stem Cell Bank, Chinese Academy of Sciences) were cultured in DMEM (Gibco) supplemented with 10% horse serum (Gibco) and 1% penicillin-streptomycin (Gibco). J774A.1 cells (purchased from iCell Bioscience Inc.) were cultured in DMEM (Gibco) supplemented with 10% fetal bovine serum (FBS, Bovogen) and 1% penicillin-streptomycin (Gibco). All cells were incubated at 37°C and 5% CO2 atmosphere. Further, all cells were tested by MycoBlue *Mycoplasma* Detector (Vazyme) to confirm the absence of *mycoplasma* contamination.

### *In vitro* studies

For biological validation, the mesh samples were placed in multi-wells plates (24 wells). The mesh samples were cut into 1 cm square. Prior to evaluation, all samples were sterilized by plasma sterilization. Cells were first counted and seeded with approximately 80,000 cells per well in a 24-well cell culture plate (Corning). The meshes and cells were co-cultured and set up in 3 biological replicates for 1, 3 and 5 days, respectively.

### Cell viability/cytotoxicity assays

The Cell Counting Kit 8 (CCK8) assay (Yeasen) was employed to determine the viability of different cells after co-culture with different meshes. The mesh samples and suspensions were removed and then 550 μL of CCK-8 reaction solution (10% CCK-8) was added to each well and incubated in a cell incubator for 4 h. The suspension was transferred to a 48-well plate and the absorbance of the liquid was measured at 450 nm using a microplate reader (Synergy2). Cell viability was calculated using the following formula, Cell viability = (sample group-B)/(C-B) ×100%, where sample groups are different meshes. B represents blanks. C represents controls.

### Live/dead cell staining assays

After removing the mesh samples the cells were washed with 1X PBS buffer and then stained using the Calcein-AM/PI double staining kit (Yeasen) according to the manufacturer’s instructions. 200 ul staining solution (final concentration of 2 μM Calcein-AM and 4.5 μM PI) was added to each well and incubated in a cell incubator for 15 min. Afterward, the stained cells were rinsed with 1X PBS buffer and then examined with an inverted microscope. Photographs were obtained under a fluorescence microscope (model) using emission filters of 490 ± 10 and 545 nm, respectively.

### Cell cytoskeleton assay

The mesh samples were removed, the cells were then fixed with 1X PBS buffer containing 4% formaldehyde (without methanol) for 10 min at room temperature. Cells were permeabilized with 0.5% Triton X-100 solution for 5 min at room temperature. 200 μL of prepared FITC-labeled phalloidin working solution (final concentration, 100 nM) was added to each well and incubated for 30 min at room temperature in the dark. The nuclei were counterstained with 200 µL of DAPI solution (concentration, 100 nM) for 30 s. The cells in each well were washed with 1X PBS buffer. Photographs were obtained under an inverted microscope using a FITC excitation/emission filter (Ex/Em = 496/516 nm) and a DAPI excitation/emission filter (Ex/Em = 364/454 nm), respectively.

### Characterization of materials and devices

The micro-morphology of the prepared polypropylene was observed and characterized by scanning electron microscope (SEM, United States, Sirion 200 (SEM)/*/Sirion 200). The surface morphology was studied by Raman image-scanning electron microscopy (RISE) combined with a cryo-embedding cut (RISE-Magna, Czech). X-ray diffraction (D8 ADVANCE Da Vinci, Germany) was used to provide chemical information on the solid surface and interface.

### Dynamic thermomechanical analysis

A dynamic thermomechanical analyzer (Q850, United States) was used to measure the mechanical properties of the strips. Firstly, test strip specimens were cut out along the two main directions of the patch, measuring 20 × 7.5 × 0.56 mm. The temperature of the furnace was then adjusted to keep the strip in the preset temperature environment. The strip was fully stretched by pre-tensioning (0.05 N). Subsequently, the initial length of the stretch was obtained by taking images and processing them in grey scale. Previous studies demonstrated that the strain range of 0%–10% was the elastodynamic region, 54 mm/min was essentially close to the instantaneous stretching rate and 6 mm/min was regarded as the quasi-static stretching rate of the polypropylene strips. The rate of sampling points was proportional to the tensile strain rate to ensure an abundance of data points and an adequate reflection of the curve trend. Specifically, stretching rate of 6 and 54 mm/min correspondeed to sampling frequencies of 6 and 54 bpm, respectively.

### MD simulation methods

The parallel molecular dynamics code LAMMPS with region decomposition was used to analyze the polymer simulation units. The established polypropylene structures were subsequently formatted into LAMMPS data files. The molecular dynamics code was then inserted and equilibrated prior to the formal polymer calculations. CVFF force fields were used to describe the interactions between the atoms. The interactions between the titanium atoms were described by the Eam potential function. This function was optimized for the phase transition characteristics of the Ti atoms at different temperatures. And for the formation of internal lattice defects. In addition, the LJ force field was also used for the interaction potential between the Ti atoms and each atom of polypropylene. After the introduction of the polypropylene model it was first relaxed in 100,000 steps at a control temperature of 300 K under NPT system synthesis to remove pre-stress in all directions. The model was then extended by 10 nm in the *z*-direction as a space for the deposited motion of the Ti atoms. The equilibrium sequence relaxed the initial configuration of any high energy. This allowed the trajectories of the atoms to be obtained and meanwhile enabled the evolutionary features of the whole system to be observed, thus facilitating the analysis. The following parameters were used in the simulations. NPT system synthesis, Nosé-Hoover pressure regulation, temperature control and 1 fs was used as time step. The mechanical responses of the different initial configurations were averaged to accommodate the entropic effects due to the large number of possible chain configurations.

### Statistical analysis

Experimental data are expressed as mean ± standard deviation. Differences between groups were performed by an unpaired two-tailed Student’s *t*-test using Excel software (significance set at *p*-value = 0.05). Bonferroni correction was applied, resulting in a significant level set at *p* < 0.0083.

## Data Availability

The raw data supporting the conclusion of this article will be made available by the authors, without undue reservation.
