# Supplementary material for: The effect of surface nucleation modulation on the mechanical and biocompatibility of metal-polymer biomaterials
Source: Front Bioeng Biotechnol. 2023 Apr 6;11:1160351. doi: 10.3389/fbioe.2023.1160351 (PMC10117951; doi:10.3389/fbioe.2023.1160351)
Supplement: Supplementary file 1 [file DataSheet1.PDF]

Supplementary Material

The effect of surface nucleation modulation on the mechanical and biocompatibility of metal-polymer biomaterials

Zhenhong Ye[1,7], Le Zhang[2], Taiwei Liu[3], Weicheng Xuan[1], Xiaodong He[4], Changhao Hou[5], Donglin Han[1], Binbin Yu[1], Junye Shi[1], Jie Kang[6*], Jiangping Chen[1,7*]

*** Correspondence:** Jie Kang: kangjie007@163.com

1. Supplementary Figures

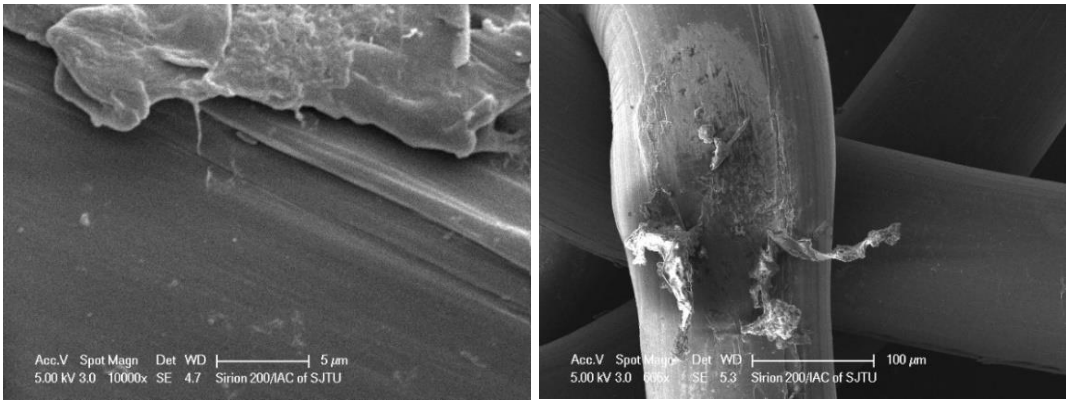

Supplementary Figure 1. Magnified local view of the polypropylene surface with irregular surface defects. Different methods of processing and synthesis of polypropylene lead to plaques and burrs, which trigger corresponding physiological inflammation and even require secondary surgical treatment in severe cases

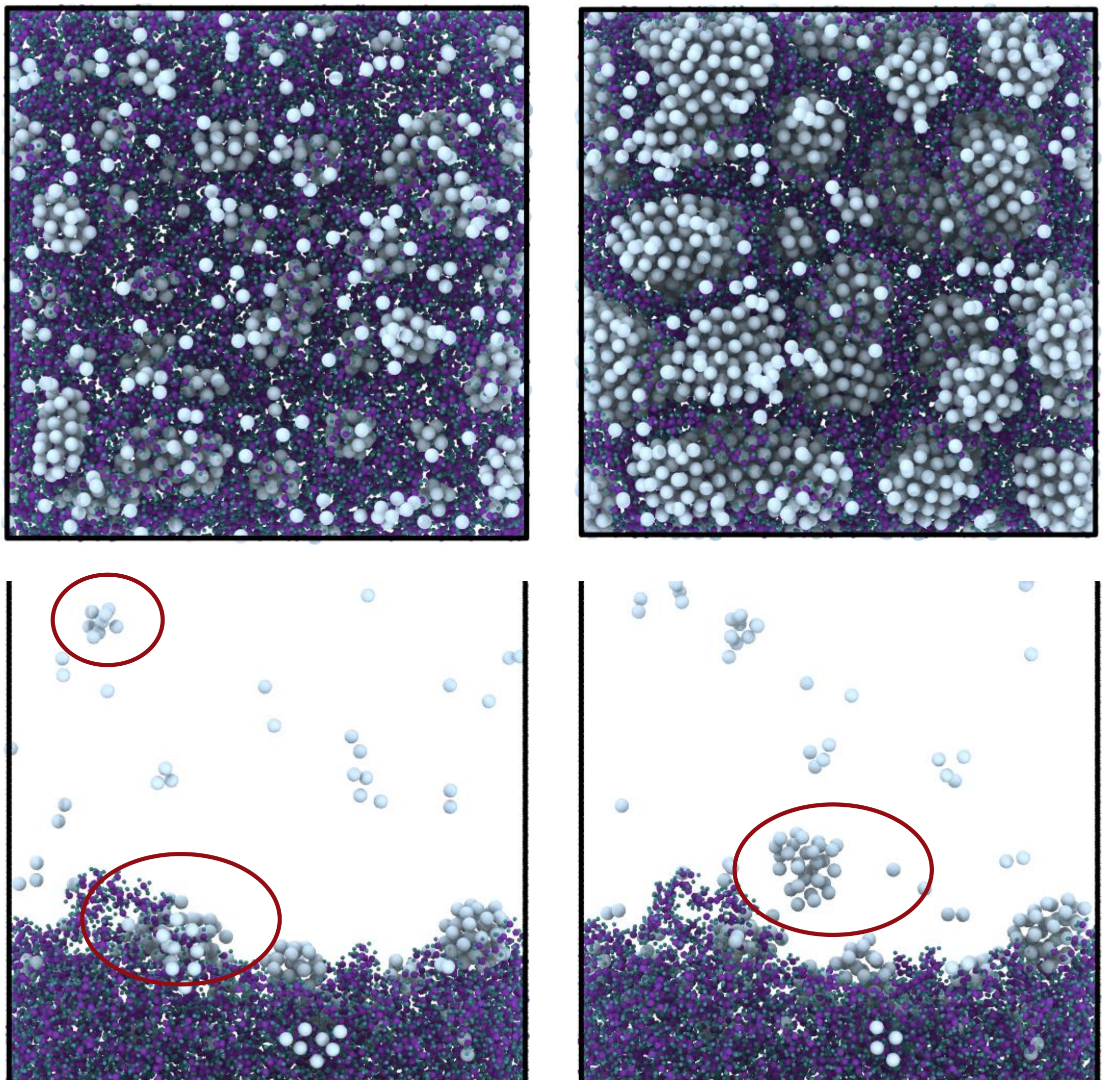

Supplementary Figure 2. The deposition of atoms and nanoclusters is accompanied by reflections and migrations, leading to an exceptionally complex overall vapor-phase deposition process. The migration at the surface is closely related to surface roughness and site potential energy.

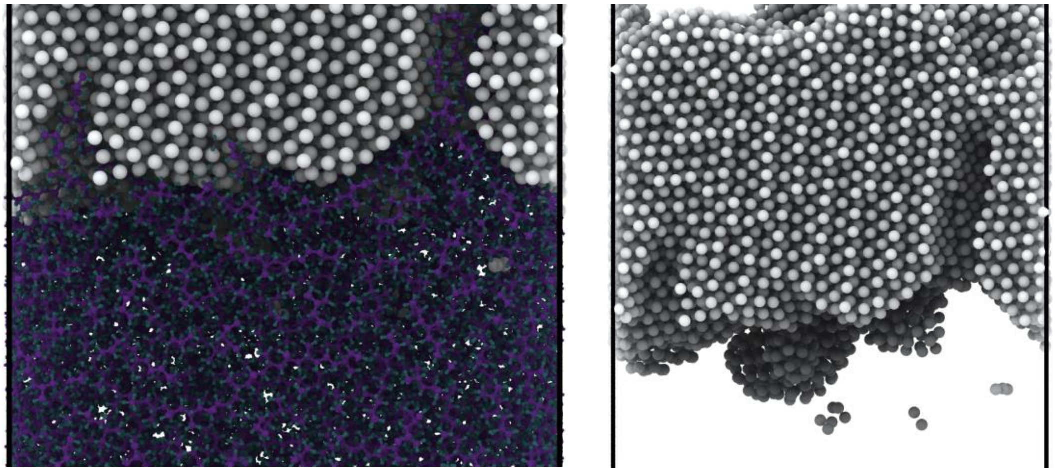

Supplementary Figure 3. Schematic diagram of Ti particle injection. The particles are free from the confinement of the nucleus and appear inside the polypropylene gap and continuously change position during relaxation.

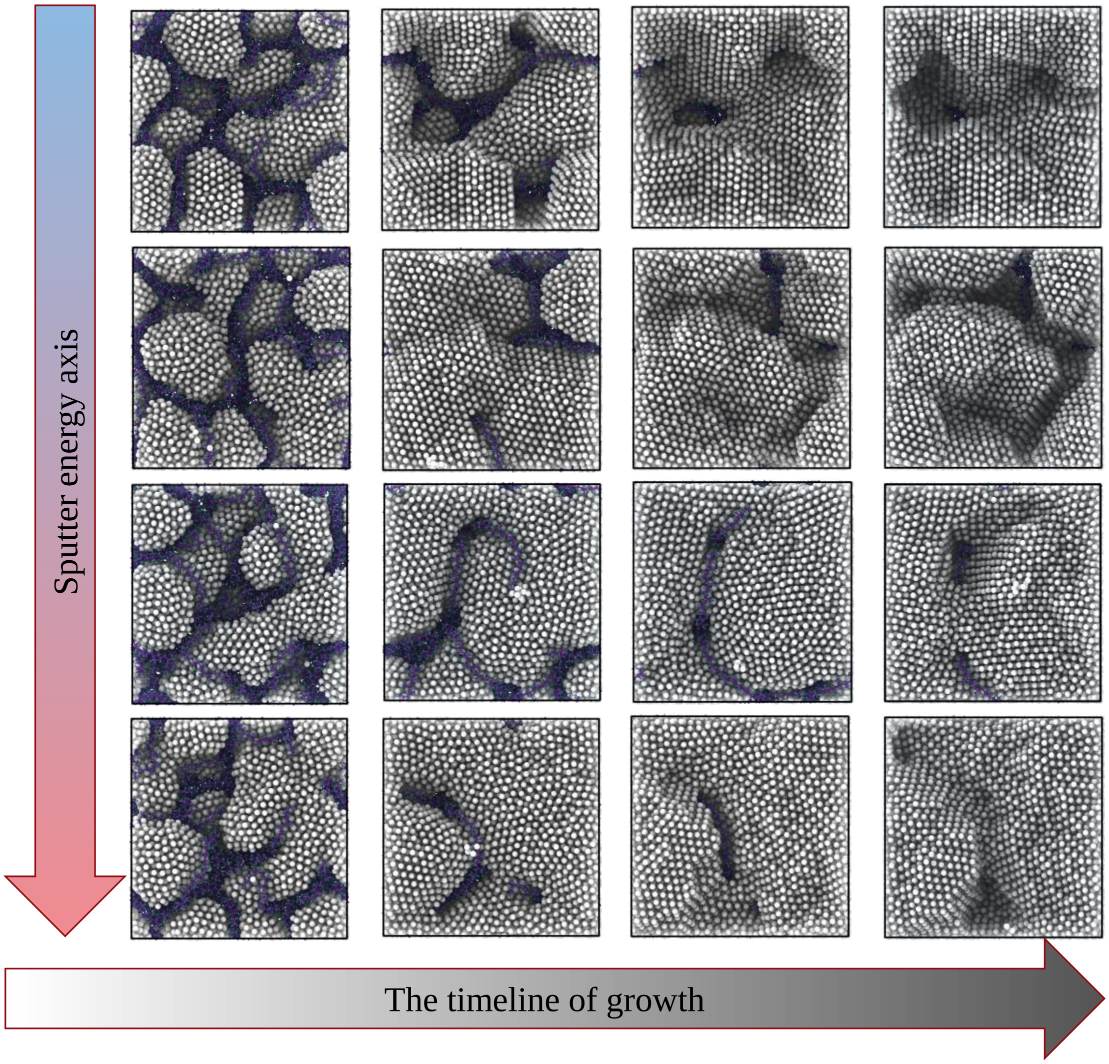

Supplementary Figure 4. The change in surface morphology with time and sputtering energy, where the gray axis represents the change in time. The atomic deposition process and is divided into four periods according to the surface morphology, which varies with sputtering time and film thickness. The energy axis is shown on the left side, where the color of the axis changes from blue to red representing the direction of gradual increase of energy (sputtering power). The roughness increases with the sputtering power due to the energized molecular chains of the polymer preventing the fusion of grain boundaries and the formation of grooves. The formation of deep grooves at higher energies leads to difficulties in the complete coverage of the polypropylene molecular chains.

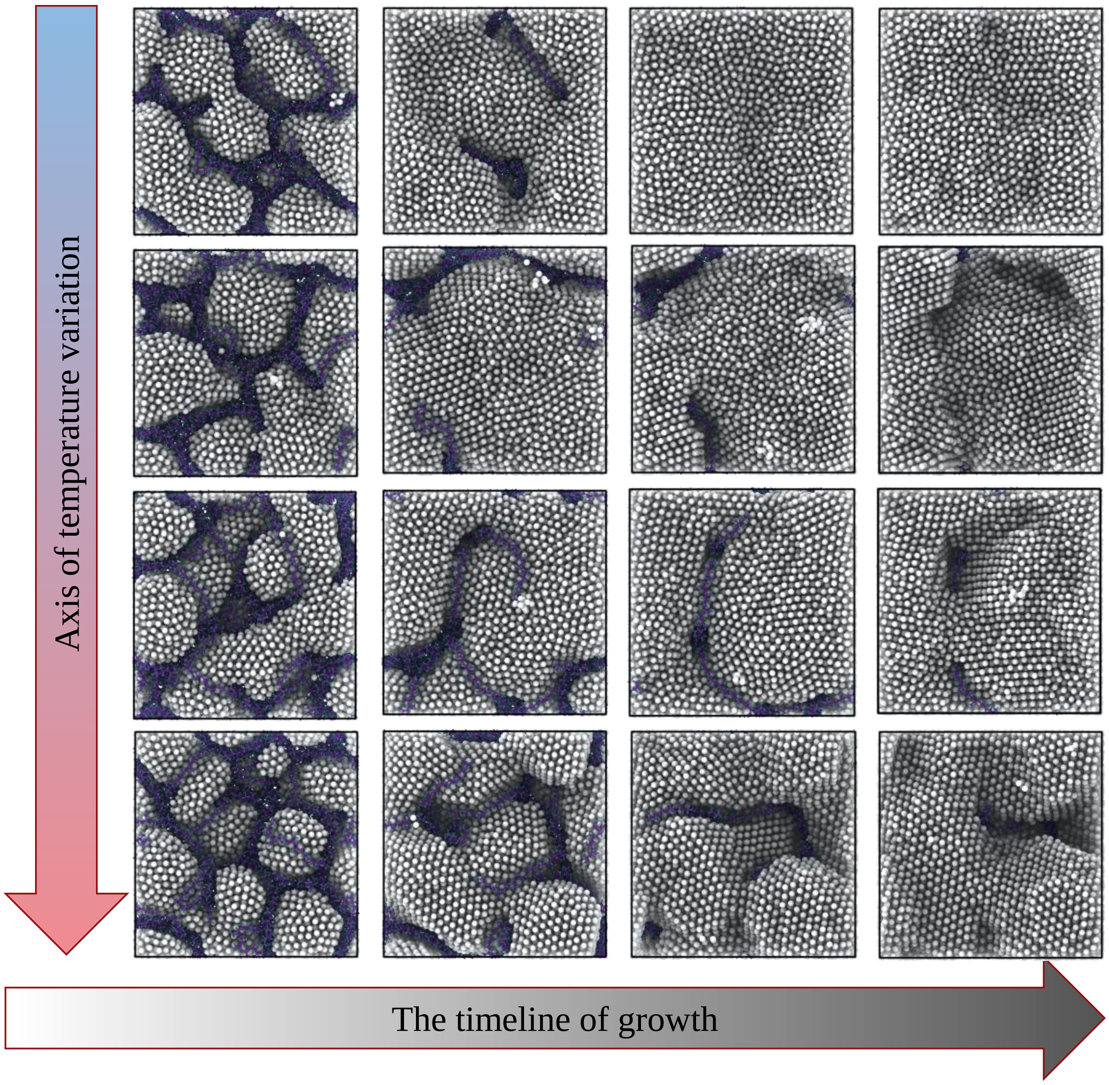

Supplementary Figure 5. Changes in the surface morphology of the coating with time and the surface temperature of polypropylene, where the gray axis represents the change in time. The temperature axis is shown on the left side, where the color of the axis changes from blue to red representing the direction of the gradual increase in temperature of the molecular chains on the surface. The roughness increases gradually with temperature in the overall trend. The molecular chains of polypropylene are quickly and completely covered at lower temperatures, and the film thickness is thin while achieving good isolation and bringing stable biocompatibility effects.

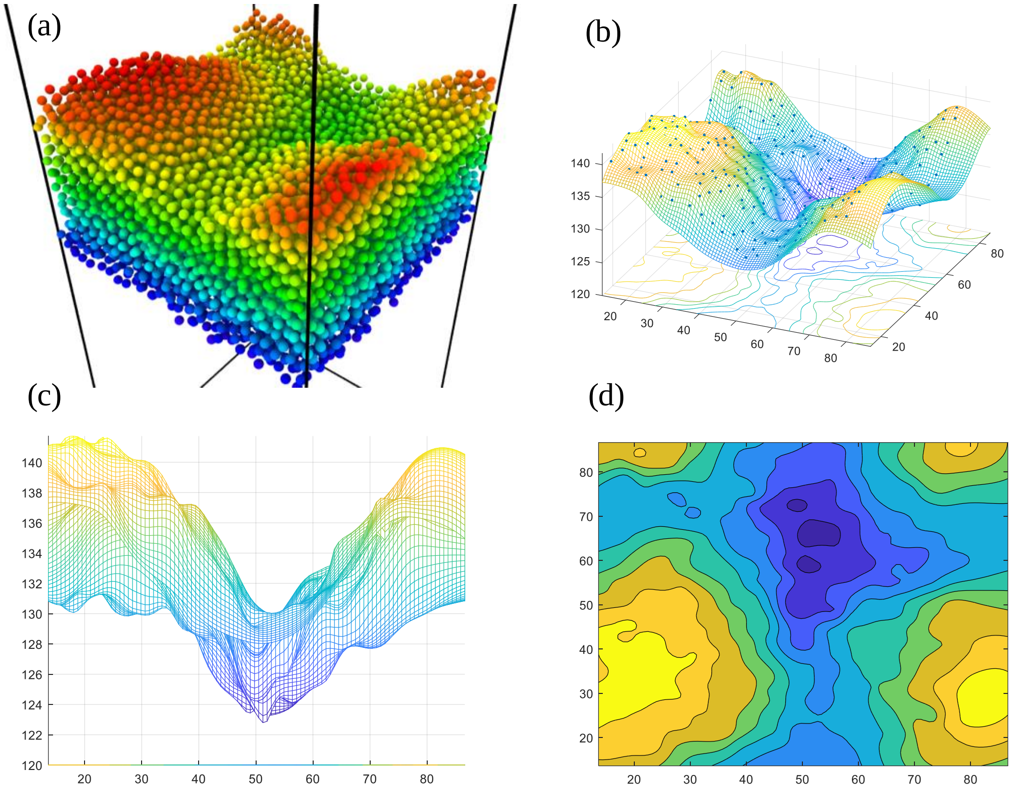

Supplementary Figure 6. (a) The position of each atom is obtained by discretizing the calculated surface morphology map; (b) All atomic sites of the surface layer are extracted to obtain the arithmetic mean deviation of the contours and the maximum height of the contours. (c) Calculation of the side view of the surface; (d) Calculation of the bottom contour map.

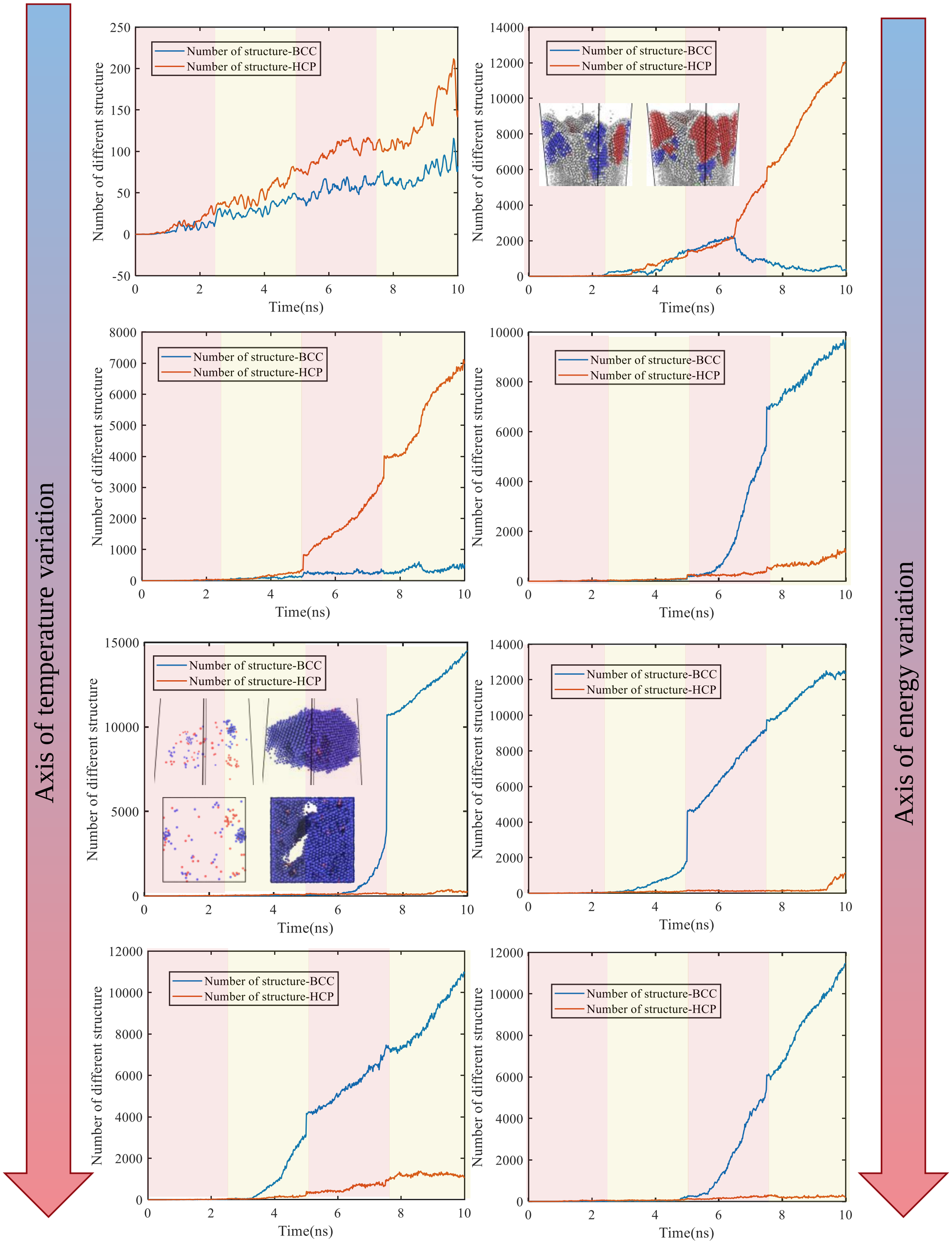

Supplementary Figure 7. (a) Schematic diagram of the crystal structure change with the increase of substrate temperature. The effect of temperature on the crystal lattice is more obvious, and the high temperature makes the atoms form a regular BCC structure faster during the deposition process. Every 2.5 ns time of deposition is carried out for a 2 ns duration, so some steps can be seen when the different color intervals are exceeded (light yellow and light red are the deposition time regions, respectively, and the junction is the relaxation region). The HCP dominates in the first two figures, and the BCC dominates as the temperature increases. (b) Schematic diagram of the crystal structure change with sputtering power. The BCC structure tends to form more stable structures due to having

lower free energy sites at different sputtering powers, when the surface temperature is fixed at 300 K. HCP prevails only when the sputtering power is highest. There is a greater tendency in the film treatment to obtain BCC layers on polypropylene for good plasticity and ductility.

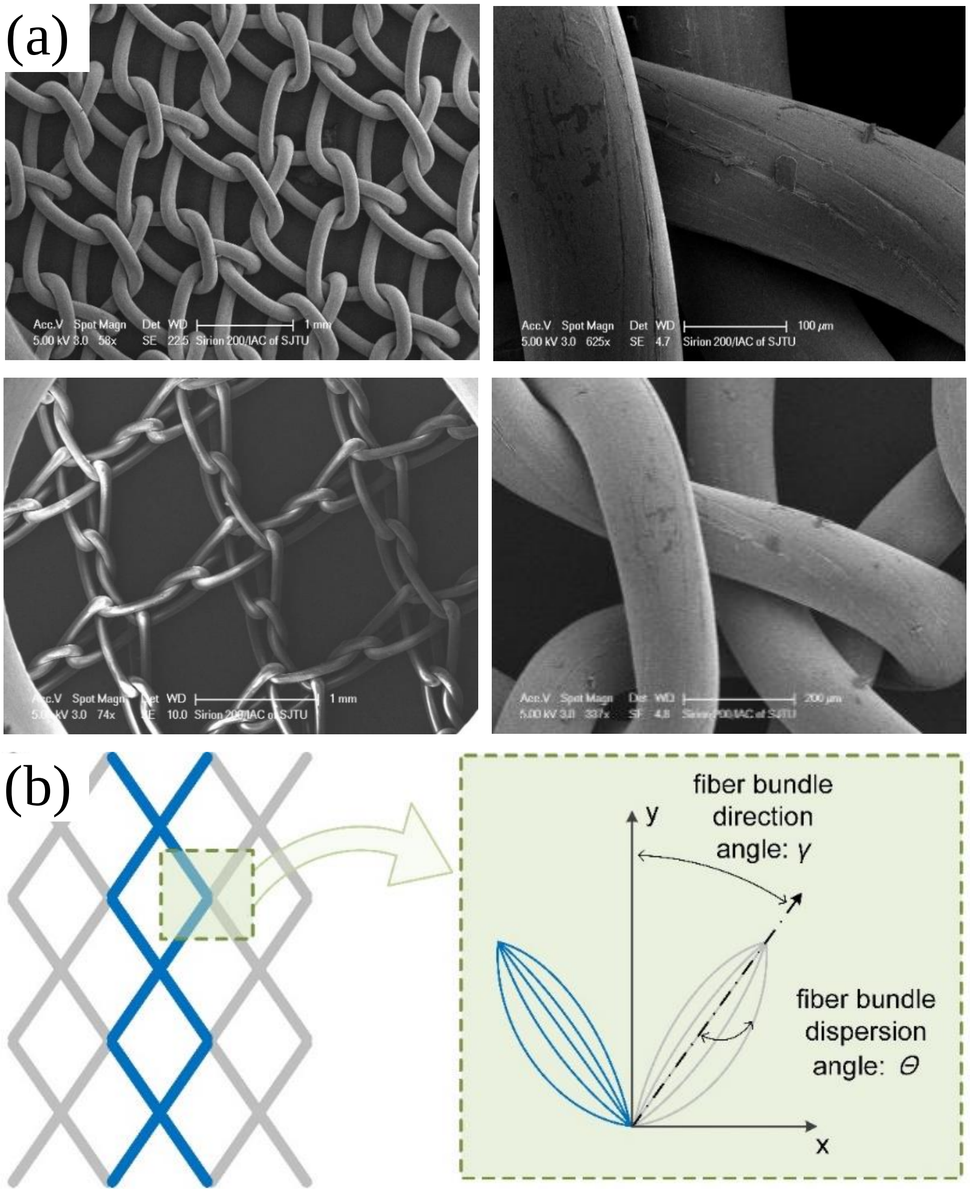

Supplementary Figure 8. (a) Microscopic enlargement of different weave types of polypropylene fibers. The first structure has close mechanical properties in the transverse and longitudinal directions and is not suitable for long service in the human abdominal wall. The second type can match well the anisotropic mechanical properties of the human abdominal wall by blending the angle of the rhombus. (b) Schematic diagram of the woven structure of the second type of polypropylene fiber, with a monofilament diameter of about 120 μm, consisting of many diamond-shaped lattices with an angle of 34.7°, which well matches the mechanical properties of human tissues.

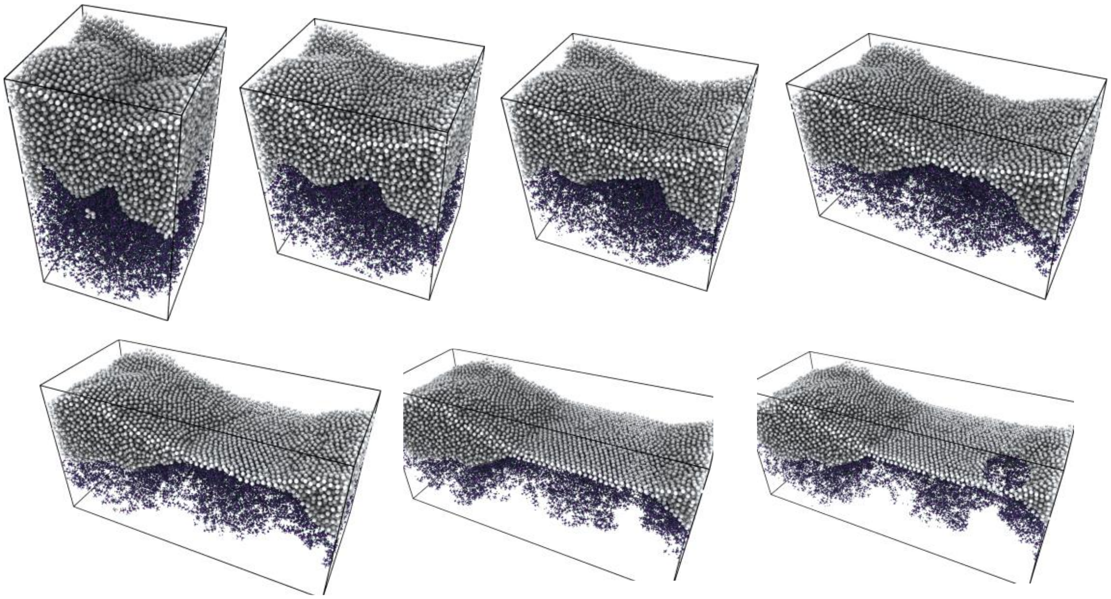

Supplementary Figure 9. Diagram of uniform film stretching and illustration of failure conditions

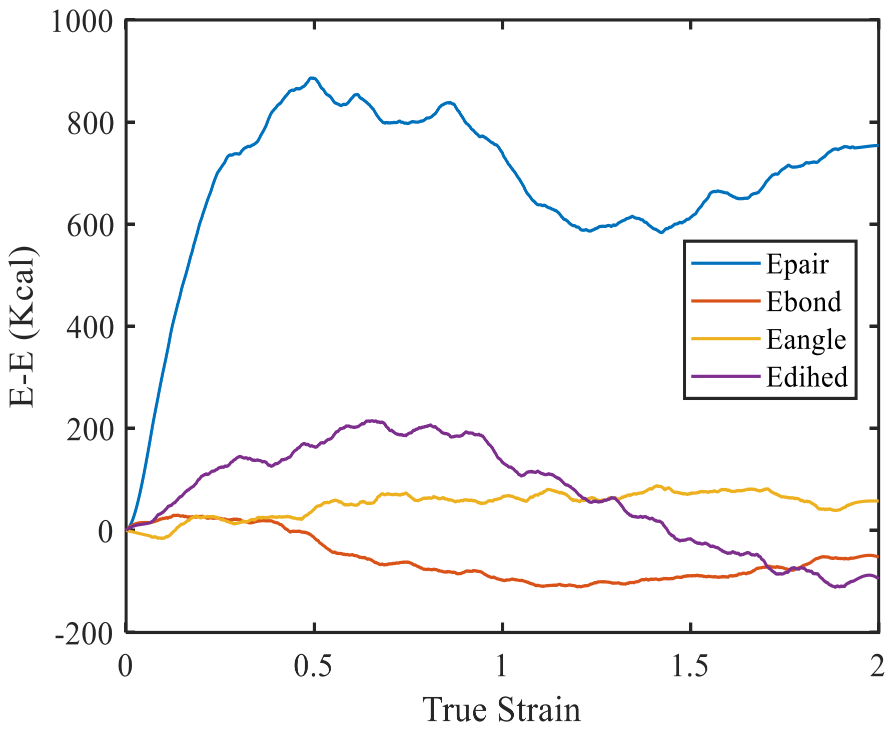

Supplementary Figure 10. Schematic diagram of molecular energy change during the stretching process

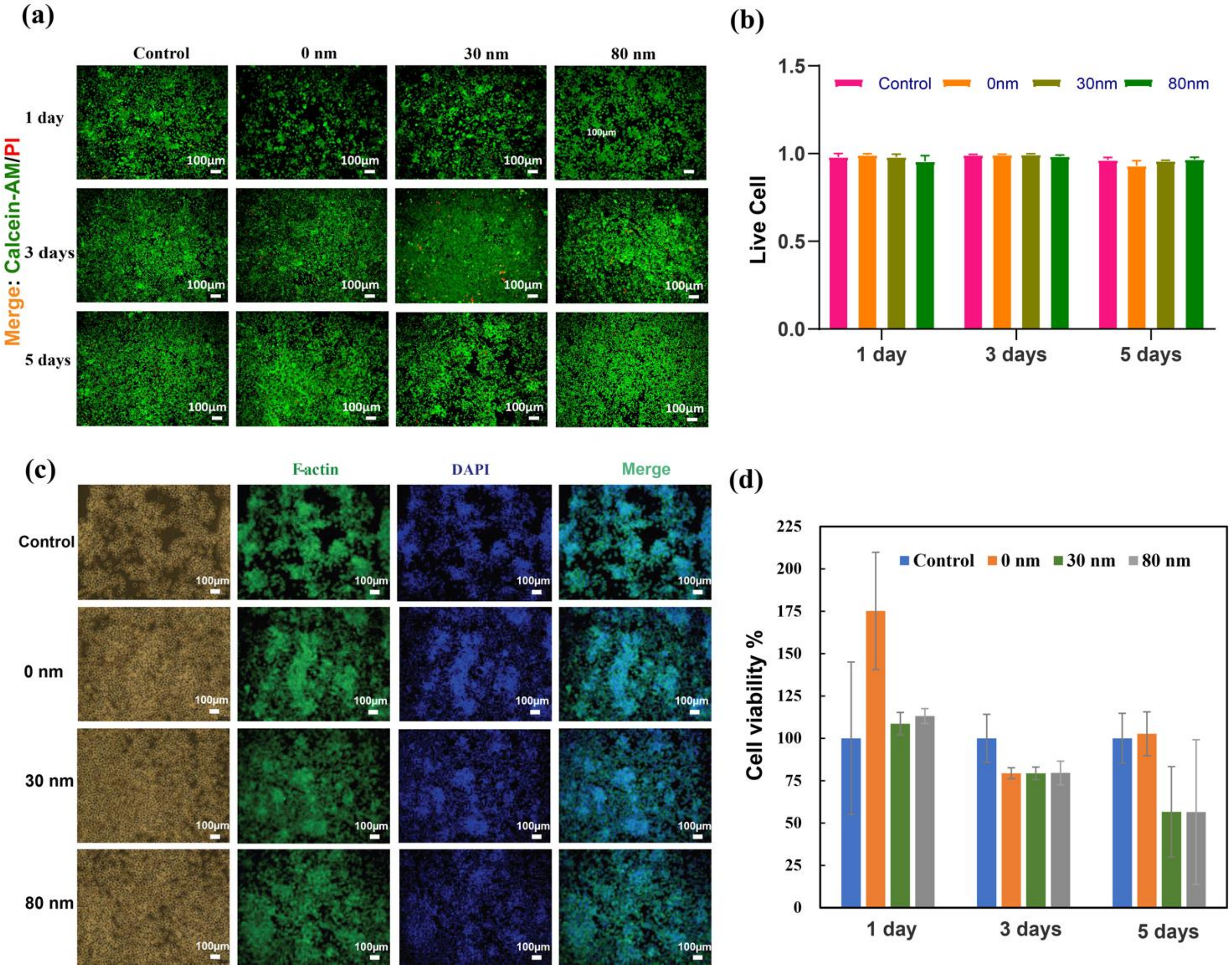

Supplementary Figure 11. In vitro biological validation with J774A.1 cells. Imaging (a) and counting (b) of live/dead cell assay using the Calcein-AM/PI double staining kit. Cytoskeleton assay with FITC-Phalloidin at day 5 (c). Cell viability/toxicity assay with the CCK8 kit (d). Different mesh samples (0, 30 and 80 nm) were co-cultured with J774A.1 cells for different periods (1, 3 and 5 days), respectively. Each bar represents the mean ± standard deviation of three biological replicates. There are no significant differences between the groups according to the t-test.

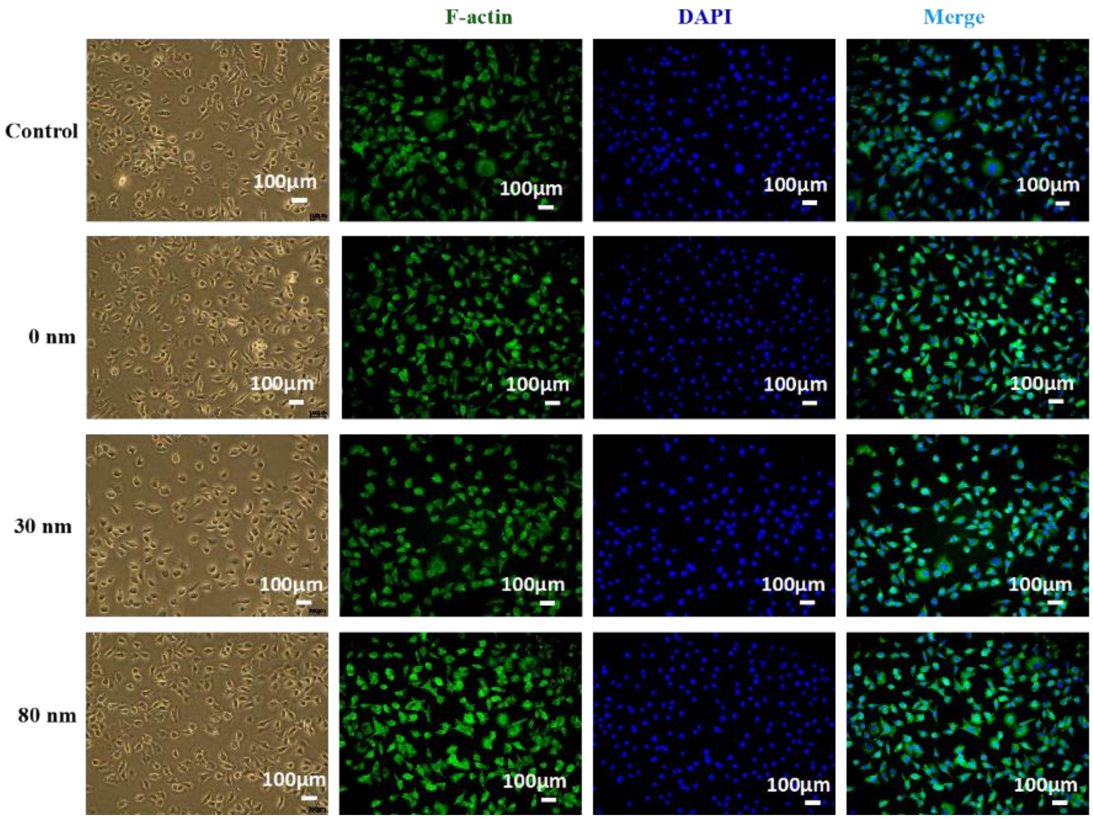

Supplementary Figure 12. Detection of the cytoskeleton by FITC-Phalloidin in L929 cell on day 1

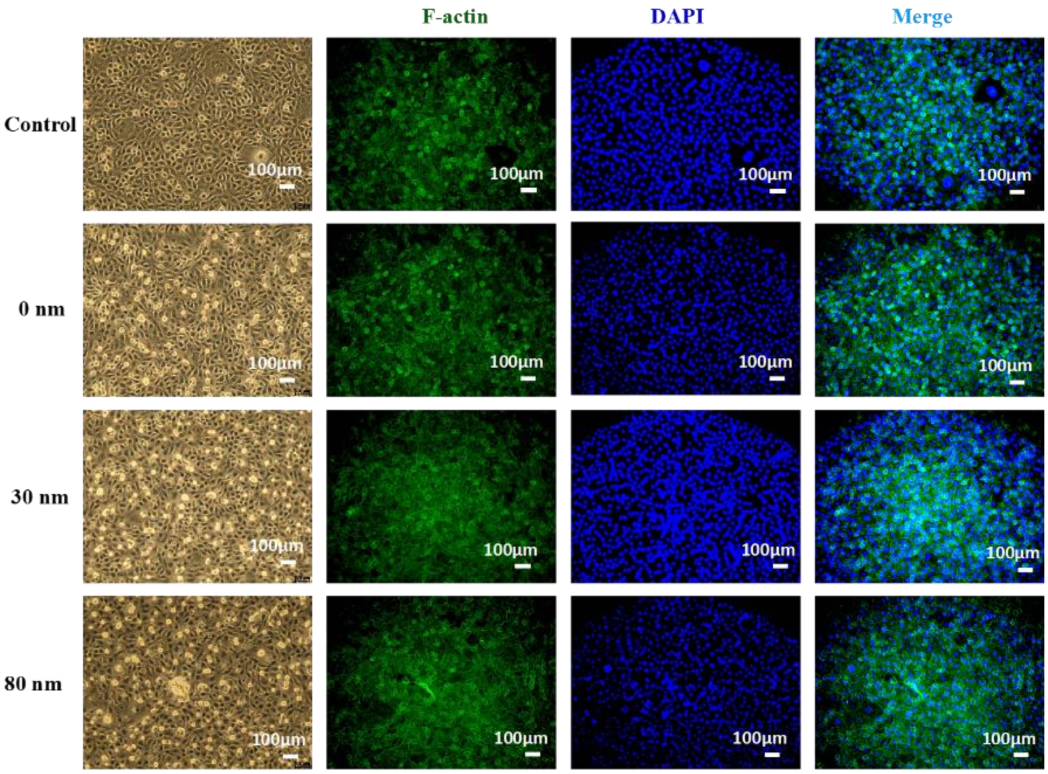

Supplementary Figure 13. Detection of the cytoskeleton by FITC-Phalloidin in L929 cell on day 3

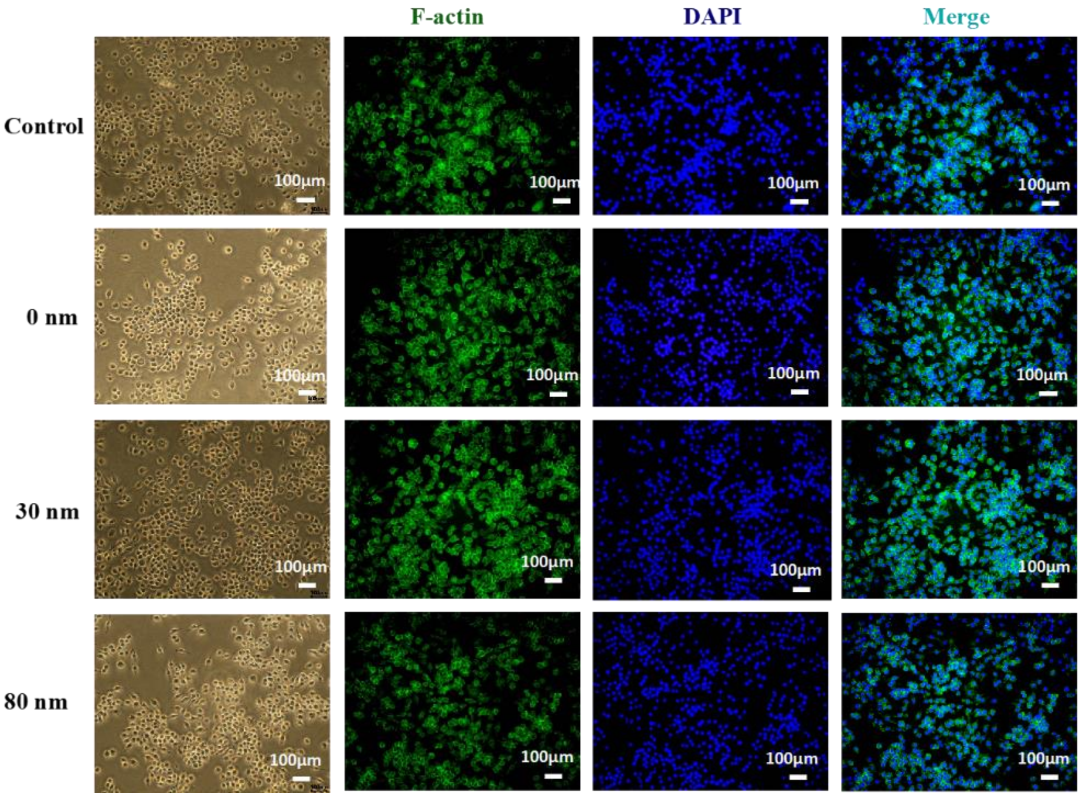

Supplementary Figure 14. Detection of the cytoskeleton by FITC-Phalloidin in J774A.1 cell on day1

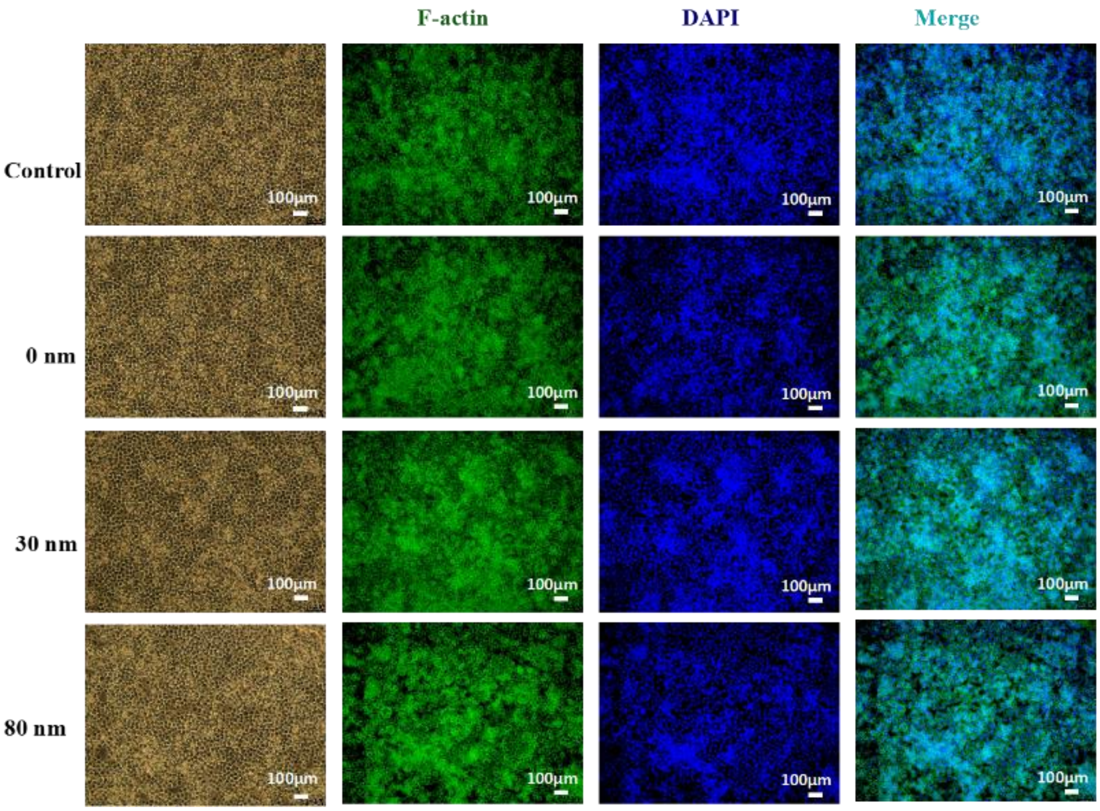

Supplementary Figure 15. Detection of the cytoskeleton by FITC-Phalloidin in J774A.1 cell on day3

2. Supplementary Note

Supplementary Note 1. Theoretical analysis

The QLV-HGO super-viscoelastic model was used to characterize the mechanical properties of the strips at different plating thicknesses and different temperatures. The strain rate information has been included in the model because of the viscoelastic effect considered in the model. Similar to the previous work, the mechanical characterization is given by fitting parameters to the tensile relaxation data of 54 mm/min. First, the stress relaxation data were extracted to obtain the QLV viscoelastic intrinsic parameters; after that, the uniaxial tensile loading data were extracted to obtain the HGO hyperelastic intrinsic parameters by combining the macro- and microstructural parameters γ and κ specific values of polypropylene strips determined from previous work. We fix the time parameters in QLV as (τ1 = 1.2 s, τ2 = 30 s, τ3 = 180 s) due to the entire stress relaxation time of 60 s in the fitting of viscoelastic intentional parameters. Due to our lack of polypropylene fiber shear data at different temperatures in the fitting of hyperelastic intrinsic parameters, we will all be given in advance in this study (C10 = 0.6178 MPa). Further, we consider that the changes in temperature and loading strain rate do not affect the polypropylene strip macro microstructure because polypropylene material is a biomaterial. The mechanical property parameters for different coating thicknesses at two temperatures are summarized in the following table. Specific details of the fitting of the present constitutive equations can be found in our previous work. Combining the super-viscoelastic parameters obtained from the 54 mm/min fit, we can obtain the mechanical behavior under 6 mm/min loading according to the QLV-HGO model. The mechanical behaviors characterized by the model fit the experimental data, indicating that our experimental and numerical results possess the classical viscoelastic physical laws.

Table 1. Constitutive parameters of the QLV-HGO model for the tensile hernia patch strips

Constitutive models	Parameters (unit)	0 nm		30 nm		80 nm	
		0 °C	37.5 °C	0 °C	37.5 °C	0 °C	37.5 °C
QLV model (τ_1 = 1.2 s, τ_2 = 30 s, τ_3 = 180 s)	g_1	0.1378	0.1136	0.0907	0.1294	0.0997	0.1469
	g_2	0.0127	0.3193	0.0888	0.1303	0.0864	0.0798
	g_3	0.4844	0.0638	0.3592	0.4891	0.0903	0.4388
	g_∞	0.3652	0.5033	0.4613	0.2512	0.7236	0.3344
	R^2	0.9995	0.9854	0.9996	0.9972	0.9997	0.9942
HGO model	C_{10} (MPa)	0.6178					
	k_1 (MPa)	1.8403	0.1071	3.446	0.4153	20.81	2.3298
	k_2	77.1602	51.8511	54.3442	94.0028	20.1271	45.8321
	R^2 (QLV-HGO model fit)	0.9464	0.856	0.8470	0.9132	0.9849	0.9118

Supplementary Note 2. Theoretical analysis of QLV-HGO visco-hyperelastic model

For characterizing hyperelastic behavior, Gasser-Ogden-Holzapfel (HGO) model can mimic the mechanical laws of the whole and internal fibers of the loading artery in multiscale[1]. Up to now, the HGO model has been usually applied in characterizing nearly incompressible soft tissue or biomaterials[2–4]. For characterizing viscous behavior, the quasi-linear viscoelastic (QLV) model has been a good choice for quantifying the viscoelasticity of kinds of materials since it came out in the last century[5–7]. QLV model can accurately capture the viscous relaxation process of materials although the model owns simple expression, and there are still many studies that favor the QLV model for viscosity characterization.

QLV model and HGO model are combined to form the QLV-HGO visco-hyperelastic model to characterize the mechanical properties of the hernia repair strip. Similar to previous related studies in visco-hyperelastic mechanical modeling, we also use the strain energy density function to expand our formula derivation process, both expression of strain energy density W and stress σ:

$$W\left(W_{\text{hyper}},t\right)=\int_0^t \beta\left(t-s\right)\frac{\partial W_{\text{hyper}}}{\partial s}\,ds \tag{1-1}$$

$$\sigma\left(t\right)=\int_0^t \beta\left(t-s\right)\left(\lambda\frac{\partial W_{\text{hyper}}}{\partial \lambda}\right)\Big/\partial s\,ds \tag{1-2}$$

where β(t) forms at the decaying exponential form of the Prony series in the QLV model that:

$$\beta(t)=g_\infty+\sum_{i=1}^{N} g_i e^{-\frac{t}{\tau_i}} \tag{2}$$

to simulate the viscous behavior of strip in stress relaxation.

For the hyperelastic response, W_{hyper} represents its strain energy density. Herein, combined with Eq. (1), W_{hyper} can be expressed as:

$$W_{\text{hyper}}=W\left(\mathbf{C},\mathbf{H}_1,\mathbf{H}_2\right) \tag{3}$$

where **C** is the right Cauchy-Green strain tensor. With the determined structural parameters, we believe that the W_{hyper} in the HGO model which is composed of tr($\mathbf{H}_1\mathbf{C}$) and tr($\mathbf{H}_2\mathbf{C}$), could characterize the strip's mechanical behavior just as

$$W_{\text{hyper}}=C_{10}\left(I_1-3\right)+\sum_{i=1}^{N=2}\frac{k_{1i}}{2k_{2i}}\left\{\mathrm{e}^{k_2\left[\mathrm{tr}\left(\mathbf{H}_i\mathbf{C}\right)-1\right]^2}-1\right\} \tag{4-1}$$

$$\mathrm{tr}\left(\mathbf{H}_i\mathbf{C}\right)=\kappa_i I_1+\left(1-3\kappa_i\right)I_{4i} \tag{4-2}$$

where I_{4i} is the fourth (i=1) and sixth (i=2) invariants whose value are equal to the square of the stretch along the i-th fiber bundle direction; N is the total number of collagen fiber bundles and polypropylene mesh is two; k_{1i} and k_{2i} are constitutive parameters for two fiber bundles. Since the mesh is woven from one kind of polypropylene material, we believe that k_{11}=k_{12}=k_1, k_{21}=k_{22}=k_2, and

κ_1=κ_2=κ. The default assumption of this model is that collagen fibers can only withstand tensile stress, and when compressed, they will become crimped and do not generate compressive stress.

Under the uniaxial tension, the horizontal and vertical stretches are λ_1 and λ_2, respectively.

$$I_{41} = \lambda_1^2 \cos^2 \gamma + \lambda_2^2 \sin^2 \gamma = I_{42} \equiv I_4 \tag{5}$$

Combining the formula above, we could simplify Eq. (4) to

$$W = C_{10}\left(I_1 - 3\right) + \frac{k_1}{k_2}\left\{ \mathrm{e}^{k_2\left[\kappa I_1 + (1-3\kappa) I_4 - 1\right]^2} - 1 \right\} \tag{6}$$

For incompressible materials, the hydrostatic pressure p is included in the Cauchy stress term. To facilitate the model characterization, we use the formula that

$$\sigma_i = \lambda_i \frac{\partial W(\lambda)}{\partial \lambda_i} - p \qquad (i = 1, 2, 3) \tag{7}$$

to highlight the p as Lagrange multiplier which can be determined by boundary conditions. We substitute Eq. (6) into Eq. (7), and obtain the detailed expressions of the three principal Cauchy stresses of the HGO model.

The prerequisite of incompressibility is enforced on polypropylene mesh hernia patches in advance. So, we use

$$\lambda_1 \lambda_2 \lambda_3 = 1 \tag{8}$$

to reduce the independent variable λ_i. This approximation allows the expression with a 3-dimensional tensor to be derived into the unidirectional vector form, and the constitutive parameters could be regressed according to the experimental data. We take Eq. (7) and (8) into account and finally obtain relationships that:

$$\sigma_{11} = 2C_{10}\left(\lambda_1^2 - \lambda_1^{-2}\lambda_2^{-2}\right) + 4k_1 \alpha e^{k_2 \alpha^2} \left\{ \kappa\left(\lambda_1^2 - \lambda_1^{-2}\lambda_2^{-2}\right) + (1-3\kappa)\lambda_1^2 \cos^2 \gamma \right\} \tag{9-1}$$

$$0 = 2C_{10}\left(\lambda_2^2 - \lambda_1^{-2}\lambda_2^{-2}\right) + 4k_1 \alpha e^{k_2 \alpha^2} \left\{ \kappa\left(\lambda_2^2 - \lambda_1^{-2}\lambda_2^{-2}\right) + (1-3\kappa)\lambda_2^2 \sin^2 \gamma \right\} \tag{9-2}$$

$$\alpha = \kappa\left(\lambda_1^2 + \lambda_2^2 + \lambda_1^{-2}\lambda_2^{-2}\right) + (1-3\kappa)\left(\lambda_1^2 \cos^2 \gamma + \lambda_2^2 \sin^2 \gamma\right) - 1 \tag{9-3}$$

By means of the finite time increment formula, we give the discrete form of the viscoelastic numerical integration that:

$$\sigma\left(t_{\mathrm{n+1}}\right) = g_\infty \sigma_{11}\left(\lambda_1\right) + \sum_{\mathrm{i=1}}^{\mathrm{N}} h_{\mathrm{i}}\left(t_{\mathrm{n+1}}\right) \tag{10-1}$$

$$h_{\mathrm{i}}\left(t_{\mathrm{n+1}}\right) = e^{-\Delta t/\tau_{\mathrm{i}}} h_{\mathrm{i}}\left(t_{\mathrm{n}}\right) + g_{\mathrm{i}} \frac{1 - e^{-\Delta t/\tau_{\mathrm{i}}}}{\Delta t/\tau_{\mathrm{i}}} \left\{ \sigma_{11}\left(\lambda_1[\mathrm{n+1}]\right) - \sigma_{11}\left(\lambda_1[\mathrm{n}]\right) \right\} \tag{10-2}$$

coupling with the hyperelastic tensile model of the Eq. (9), the final form of the unidirectional QLV-HGO visco-hyperelastic constitutive model is obtained.

With the help of the Python code under the Pycharm development environment, we have calibrated and verified the QLV model, the HGO model, and the QLV-HGO finite time increment formula related in this study. Leastsq function from scipy.optimize package is imported to conduct the least square method. Since the λ_2 could not be expressed directly through the Eq. (9-2) because of implicitly embedding, we decided to approximate it numerically as follows: According to the experimental data of the tensile stretch λ_1, linear secant regression on the Eq. (9-2) (where the hydrostatic pressure p is replaced with the help of Eq. (9-3)) was performed to obtain the array of λ_2.

To evaluate the goodness of the fitted constitutive model, we calculated the total departure S_{r}, residual error S_{err}, and goodness of regression R^2 according to:

$$S_r = \sum_{i=1}^{m} (Y_i - \overline{Y})^2 \tag{11-1}$$

$$S_{err} = \sum_{i=1}^{m} (Y_i - Y_i)^2 \tag{11-2}$$

$$R^2 = 1 - \frac{S_{err}}{S_r} \tag{11-3}$$

where Y_i is the experimental data value, $\overline{Y}$ is the mean of the former all, Y_i represent the value corresponding to the fitted constitutive model, m is the number of the data pairs on fitting.

Combining with the stress relaxation experimental data of normalized Cauchy stress and time, the viscous constitutive parameters of g_1, g_2, and g_3 are obtained by using the least squares through fitting Eq. (2); Similarly, combining with the tensile data of tensile Cauchy stress (all peak stresses during ramp rest), tensile stretch and stretch rate, fiber dispersion coefficient κ (calibrated according to Method 2.1 and listed in Table 1 for specific value), and the regressed second principal stretch λ_2, the hyperelastic constitutive parameters of k_1 and k_2 are obtained by using the least squares through fitting Eq. (9-1).

Supplementary Note 3. Tensile test at low temperature

The experimental results of stress relaxation and uniaxial tension of hernia patches at -80°C are shown in the following figures. Similarly, we characterize and analyze the experimental data using the QLV-HGO intrinsic model under the time increment law to obtain the super viscoelasticity numerical model parameters as shown in Table 2. The results of this paper fully demonstrate the super-viscoelastic mechanical properties of the hernia patch strips at -80°C, and the results are convincing. Under the ultra-low temperature condition, the strip viscous properties decrease substantially and the stiffness increases substantially. Likewise, the plating condition gives a biomechanical effect of increased stiffness and reduced viscosity of the strip, which confirms our temperature hypothesis conjecture.

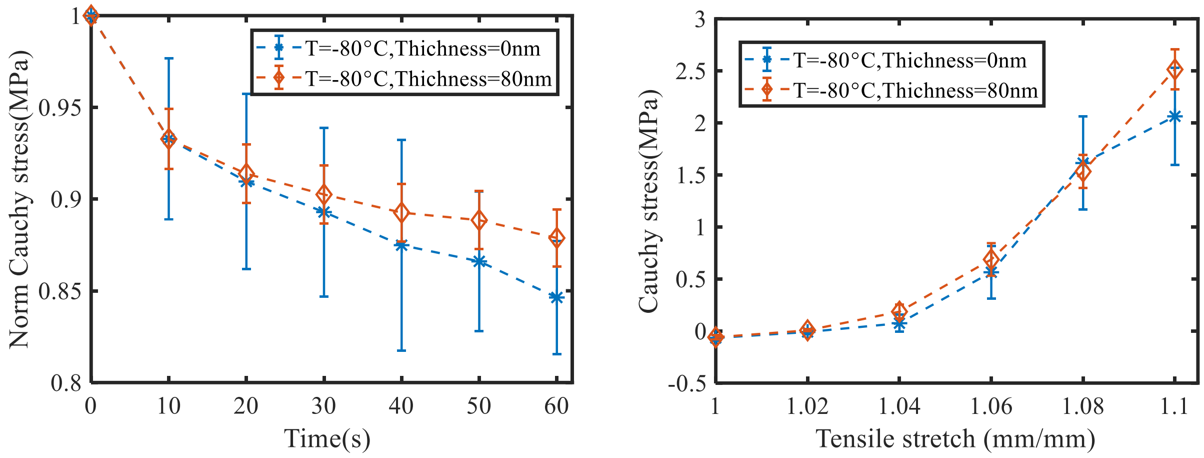

Supplementary Figure 16. Mechanical properties and characterization of the super-viscoelasticity of hernia patch strips under different plating treatments at -80°C and 5.4/min strain rate stretching: (a) tensile super-elasticity of hernia patches under normal and 80 nm plating; (b) stress relaxation viscoelasticity of hernia patches under normal and 80 nm plating.

Table 2. Verification with 0 and 80nm strips.

Constitutive models	Parameters (unit)	0 nm	80 nm
		25 °C	25 °C
QLV model (τ_1 = 1.2 s, τ_2 = 30 s, τ_3 = 180 s)	g_1	0.0435	0.045
	g_2	0.033	0.0739
	g_3	0.2797	0.038
	g_∞	0.6437	0.8431
	R^2	0.9982	0.9989
HGO model	C_{10} (MPa)	0.6178	
	k_1 (MPa)	12.0931	16.6612
	k_2	32.1287	33.3697
	R^2 (QLV-HGO model fit)	0.8815	0.9149
